# ﻿Biotrophic and saprophytic fungi from the *Rhodocybe*-*Clitopilus* clade (Entolomataceae): two new species and one new record in subtropical China

**DOI:** 10.3897/mycokeys.116.148775

**Published:** 2025-04-24

**Authors:** Sipeng Jian, Xia Chen, Tianwei Yang, Xinjing Xu, Feng Gao, Yiwei Fang, Jing Liu, Chunxia Zhang

**Affiliations:** 1 Yunnan Institute of Tropical Crops, Jinghong, Yunnan, 666100, China Yunnan Institute of Tropical Crops Jinghong China; 2 The Administration Bureau of Dr. Sun Yat-sen’s Mausoleum, Nanjing, Jiangsu, 210014, China The Administration Bureau of Dr. Sun Yat-sen’s Mausoleum Nanjing China

**Keywords:** Biotrophic species, Entolomataceae, morphology, multigene phylogeny, plant pathogens, taxonomy

## Abstract

This study proposes two new species and a new record in the *Rhodocybe-Clitopilus* clade, based on comprehensive morphological and molecular analyses. The nuc rDNA internal transcribed spacer region ITS1-5.8S-ITS2 (ITS), the large subunit ribosomal RNA gene (LSU), the RNA polymerase II second largest subunit (*RPB2*) and the translation elongation factor 1-alpha gene (*TEF1*), were employed to elucidate the relationships of *Clitopilus* and *Rhodocybe*. The first species, *Clitopilusparasiticus*, is capable of infecting the leaves of host plants in the genera *Dryopteris* and *Oplismenus*, exhibiting typical biotrophic behaviour while also demonstrating saprophytic growth on soil. Intraspecific comparisons were conducted, examining environmental factors as well as macro- and microscopic characteristics amongst individuals found on different plant hosts. Furthermore, this study reports the new saprophytic species, *Rhodocybezijinshanensis* and provides a detailed description of *Clitopilusbaronii*, a newly-recorded species in China.

## ﻿Introduction

In nature, numerous fungi are well-known for their parasitic relationships, enabling them to thrive in dynamic environments. For example, the ergot (*Clavicepspurpurea* (Fr.) Tul.) and corn smut (*Mycosarcomamaydis* (DC.) Bref.) are recognised as pathogenic fungi affecting cultivated plants (*Triticumaestivum* L. and *Zeamays* L., respectively) (Tudzynski and Scheffer 2004; McTaggart et al. 2016). Additionally, several special form genera, such as *Asterophora* Ditmar, *Squamanita* Imbach and *Hypomyces* (Fr.) Tul. & C. Tul., exhibit fungicolous parasitism or mycoparasitic behaviour (Rogerson and Samuels 2018; Elkhateeb and Daba 2021; Liu et al. 2021). However, parasitic forms are relatively rare in Agaricales Underw., particularly for biotrophic parasitism.

Saprophytic and symbiotic modes of nutrition are predominant amongst fungi in Basidiomycetes, but some fungi also employ parasitic nutrition as a strategy for survival and reproduction (Põlme et al. 2021; Shi et al. 2023). Thereinto, biotrophic parasitism is an intriguing and unique phenomenon in fungi, defined as a nutritional strategy where fungi derive nutrients from a living host while keeping it alive, often causing restricted damage to the host plant (Luttrell 1974; Kemen and Jones 2012). Species within the Agaricales that exhibit distinct biotrophic capabilities often also possess saprophytic abilities, indicating that they are not obligate parasites. For example, Zhang et al. (2022) identified a new species, viz. *Crepidotusherbaceus* T. Bau & Y.P. Ge, which is not only parasitic on the leaves or stems of *Oreocnidefrutescens* (Thunb.) Miq. and *Alpiniajaponica* (Thunb.) Miq., but is also found on the plant debris or humus.

In the family Entolomataceae Kotl. & Pouzar, there are two main clades: *Entoloma* (Fr.) P. Kumm. and *Rhodocybe-Clitopilus* (Co-David et al. 2009; Baroni and Matheny 2011; Kluting et al. 2014). The *Rhodocybe-Clitopilus* clade differs from the *Entoloma* clade by its basidiospores, which are characterized by either longitudinal ridges or scattered, finely to distinctly pustulate ornamentations (Baroni 1981; Singer 1986; Kluting et al. 2014). Within the *Rhodocybe-Clitopilus* clade, most species are primarily regarded as saprophytic (Sanchez-Garcia and Matheny 2017). However, a few species, such as *Rhodophanastangliana* (Bresinsky & Pfaff) Vizzini, *Clitopiluspasseckerianus* (Pilát) Sing., *C.fasciculatus* Noordel. and *C.daamsii* Noordel., also appear mycoparasite (Noordeloos 1984, 1993; Læssøe and Rosendahl 1994; Czederpiltz et al. 2001). Notably, *C.hobsonii* (Berk.) P.D. Orton has been reported to grow on stumps, fallen logs, twigs and living herbaceous leaves and stems (Orton 1960; Noordeloos 1984), highlighting its saprophytic and biotrophic capacities.

In the current study, several specimens gathered from Jiangsu Province are examined carefully. Three samples closely resembled *Pleurotus* (Fr.) P. Kumm., *Crepidotus* (Fr.) Staude and *Omphalotus* Fayod. Upon microscopic examination, they were all confirmed to the *Rhodocybe-Clitopilus* clade, respectively. Furthermore, two new species and one new record species were identified, based on the multi-gene phylogenetic tree. Therefore, all three species are described herein.

## ﻿Materials and methods

### ﻿Sample collections and morphological observations

The collection information of voucher specimens and the sequences used in phylogenetic analyses are shown in Table 1. The colour codes (hex triplets) from ColorHexa (https://www.colorhexa.com) were employed to depict the colour of basidiomata. These codes consist of characters ranging from a to f and 0 to 9, with each pair corresponding to the red, green and blue components of the colour. The general description of basidiomata, including both macro- and microscopic features, as well as the morphological classification rules in *Clitopilus* and *Rhodocybe* Maire were based on the work of Baroni (1981) and Jian et al. (2020a). All voucher specimens have been deposited at the Cryptogamic Herbarium of the Herbaria of Kunming Institute of Botany, Chinese Academy of Sciences (KUN-HKAS).

**Table 1.** Sequencing primers and the best annealing temperature for ITS, LSU, *RPB2* and *TEF1*.

Primer name	Nucleotide sequence 5’-3’	PCR annealing temperature (°C)
ITS4	TCC TCC GCT TAT TGA TAT GC	52
ITS5	GGA AGT AAA AGT CGT AAC AAG G	
LROR	ACC CGC TGA ACT TAA GC	52
LR5	TCC TGA GGG AAA CTT CG	
EF1-983F	GCY CCY GGH CAY CGT GAY TTY AT	56/touchdown*
EF1-1953R	CCR GCR ACR GTR TGT CTC AT	
bRPB2-6F	TGG GGY ATG GTN TGY CCY GC	52
bRPB2-7.1R	CCC ATR GCY TGY TTM CCC ATD GC	

Notes: * the details of the touchdown method refer to Kluting et al. (2014).

Sections of dried basidiomata were rehydrated in purified water and 5% potassium hydroxide (KOH) and were occasionally stained with 1% Congo Red to enhance visibility. The notation “[*n*/*m*/*p*]” indicates *n* basidiospores from *m* basidiomata of *p* specimens. The measurements of basidiospores are presented in the format (a–)b–c(–d), where the range b–c includes at least 90% of the measured values, while a and d (given in parentheses) represent the extreme values. The average length and width of basidiospore (± standard deviation) are denoted as L_m_ and W_m_, respectively. The term Q refers to the “length/width ratio” of a basidiospore in side view, with Q_avg_ representing the average Q across all specimens (± standard deviation). Fragments isolated from specimens were attached to aluminium stubs using double-sided adhesive tape, and then coated with gold/palladium. Finally, a ZEISS EVO LS10 (Germany) scanning electron microscope (SEM) was used to observe the ornamentation of the basidiospores.

The genetic names appeared in this study are abbreviated as follows: *Clitopilus* = “*C.*”, *Rhodocybe* = “*R.*”.

### ﻿Molecular phylogenetic analyses

In this study, we utilised two sequences of non-protein-coding and two protein-coding genes: the nuc rDNA internal transcribed spacer region ITS1-5.8S-ITS2 (ITS), the large subunit ribosomal RNA gene (LSU), the RNA polymerase II second largest subunit (*RPB2*) and the translation elongation factor 1-alpha gene (*TEF1*). The ITS and LSU genes were selected for their availability of universal primers (White et al. 1990), while the *RPB2* and *TEF1* genes were chosen due to their relatively high number of informative sites and sufficient nucleotide variation, which are essential for inferring evolutionary relationships within the Entolomataceae (Matheny et al. 2007; Co-David et al. 2009; Kluting et al. 2014). All the sequences were submitted to the National Center for Biotechnology Information (NCBI) and detailed information regarding each gene was provided in Table 1.

Genomic DNA was extracted from collected materials and herbarium specimens using the CTAB (cetyltrimethylammonium bromide) procedure outlined by Doyle and Doyle (1987). The PCR protocol followed the touchdown method described by Kluting et al. (2014), with detailed data provided in Table 2. Gel extraction and PCR (polymerase chain reaction) were conducted to purify the PCR products, which were then sequenced on an ABI-3730-XL sequence analyser (Applied Biosystems, Foster City, CA) using the same primers as in the PCR. The new sequences generated from this study are highlighted in bold in Table 1.

**Table 2.** Collection information of voucher specimen and GenBank accession numbers for sequences used in phylogenetic analyses. H in parentheses means the holotype specimen.

Species	Collection or collector no.	Location and year	GenBank accession numbers				References
			ITS	LSU	*RPB2*	*TEF1*	
*C.abprunulus*	KUN-HKAS 107040^a^	Macedonia 2019	NR_172792	NG_074438	MT349666	MT349670	Jian et al. (2020b)
*C.abprunulus*	KUN-HKAS 107041^a^	Macedonia 2019	MT345049	MT345054	MT349667	MT349671	Jian et al. (2020b)
*C.abprunulus*	KUN-HKAS 107042^a^	Macedonia 2019	MT345047	MT345052	MT349665	MT349669	Jian et al. (2020b)
*C.abprunulus*	MEN 2003-09-14^b^	Belgium 2003	KR261096	GQ289149	GQ289221	–	Co-David et al. (2009)
*C.albidus*	CAL 1319^c^	Kerala State, India 2001	MF926596	MF926595	MF946579	–	Raj and Manimohan (2018)
*C.amygdaliformis*	KUN-HKAS 60406^a^	Yunnan, China 2008	MN061292	–	MN148120	–	Jian et al. (2020a)
*C.amygdaliformis*	KUN-HKAS 81125^a^	Yunnan, China 2014	NR_172768	MN065681	MN148119	MN166231	Jian et al. (2020a)
*C.amygdaliformis*	KUN-HKAS 87950^a^	Yunnan, China 2014	MN061290	MN065680	MN148118	MN166230	Jian et al. (2020a)
“C.cf.argentinus”	MTB 4804/2^d^	Germany 2011	–	–	KC816907	KC816823	Kluting et al. (2014)
*C.austroprunulus*	MEN2009001^e^	Tahune, Australia 2009	KC139084	–	–	–	Crous et al. (2012)
*C.austroprunulus*	MEN2009062^e^	Tasmania, Australia 2009	KC139085	–	–	–	Crous et al. (2012)
***C.baronii***	**KUN-HKAS 145333**	**Jiangsu, China 2023**	**PQ793166**	**PQ781610**	**PQ788395**	**PQ788402**	**This study**
***C.baronii***	**KUN-HKAS 145334**	**Jiangsu, China 2023**	**PQ793167**	**PQ781611**	**PQ788396**	**PQ788403**	**This study**
*C.baronii*	K(M)179703^f^	UK 2012	MN855362	–	MN856160	–	Consiglio and Setti (2019)
*C.baronii*	AMB 18359^g^	Mantova, Italy 2006	MN855365	–	MN856163	MN856174	Consiglio and Setti (2019)
*C.baronii*	AMB 18362^g^	Ferrara, Italy 2007	MN855368	–	MN856166	MN856176	Consiglio and Setti (2019)
*C.baronii*	AMB 18363^g^	Mantova, Italy 2007	NR_176131	–	MN856167	MN856177	Consiglio and Setti (2019)
*C.baronii*	AMB 18378^g^	Pisa, Italy 2007	MN855370	–	MN856168	MN856178	Consiglio and Setti (2019)
*C.brunneiceps*	KUN-HKAS 73123^a^	Yunnan, China 2011	MN061294	MN065683	MN148122	MN166233	Jian et al. (2020a)
*C.brunneiceps*	KUN-HKAS 80211^a^	Hubei, China 2013	MN061293	MN065682	MN148121	MN166232	Jian et al. (2020a)
*C.brunneiceps*	KUN-HKAS 104510^a^	Yunnan, China 2018	NR_172769	MN065684	MN148123	MN166234	Jian et al. (2020a)
*C.brunneiceps*	HMJAU 23509^h^	Neimenggu, China 2013	MN061296	MN065685	MN148115	–	Jian et al. (2020a)
*C.chichawatniensis*	LAH37431^i^	Punjab, Pakistan 2019	ON980767	ON980764	–	–	Fatima et al. (2022)
*C.chichawatniensis*	LAH37432^i^	Punjab, Pakistan 2020	ON980766	ON980763	–	–	Fatima et al. (2022)
*C.chrischonensis*	TO HG1994^j^	Basilea, Switzerland 2008	HM623128	HM623131	–	–	Vizzini et al. (2011)
“*C.cinerascens*”	8024 TJB^d^	Florida, USA 1996	–	GU384613	KC816908	KC816824	Kluting et al. (2014)
“*C.cinerascens*”	8133 TJB^d^	Louisiana, USA 1996	–	–	KC816909	KC816825	Kluting et al. (2014)
*C.cretoalbus*	LAH37017^i^	Punjab, Pakistan 2020	OM935685	OM934826	–	–	Izhar et al. (2023)
*C.cretoalbus*	LAH35709^i^	Punjab, Pakistan 2017	ON117610	ON229505	–	–	Izhar et al. (2023)
*C.crispus*	9982 TJB^d^	Chiang Mai, Thailand 2006	–	–	KC816910	KC816826	Kluting et al. (2014)
*C.crispus*	10027 TJB^d^	Chiang Mai, Thailand 2006	–	–	KC816911	KC816827	Kluting et al. (2014)
*C.crispus*	KUN-HKAS 84667^a^	Yunnan, China 2014	MN061314	MN065705	MN148142	MN166254	Jian et al. (2020a)
*C.crispus*	KUN-HKAS 87018^a^	Yunnan, China 2014	MN061315	MN065706	MN148143	MN166255	Jian et al. (2020a)
*C.crispus*	KUN-HKAS 90506^a^	Yunnan, China 2015	MN061312	MN065702	MN148139	MN166251	Jian et al. (2020a)
*C.crispus*	KUN-HKAS 90508^a^	Yunnan, China 2015	–	MN065703	MN148140	MN166252	Jian et al. (2020a)
*C.crispus*	KUN-HKAS 97509^a^	Yunnan, China 2016	MN061318	MN065708	MN148145	MN166258	Jian et al. (2020a)
*C.crispus*	KUN-HKAS 102670^a^	Yunnan, China 2017	MN061313	MN065704	MN148141	MN166253	Jian et al. (2020a)
*C.crispus*	KUN-HKAS 104507^a^	Yunnan, China 2017	MN061316	MN065707	MN148144	MN166256	Jian et al. (2020a)
*C.cystidiatus*	MEN 200350	Slovakia 2003	–	GQ289147	GQ289220	–	Co-David et al. (2009)
*C.fasciculatus*	MO#297071	California, USA 2017	MG551863	–	–	–	Direct submission
*C.fusiformis*	SAAS 1038^k^	Yunnan, China 2015	KY385634	–	KY385632	–	Wang et al. (2017)
*C.fusiformis*	SAAS 1892^k^	Yunnan, China 2015	NR_158328	–	KY385633	–	Wang et al. (2017)
*C.fusiformis*	KUN-HKAS 104513^a^	Yunnan, China 2018	MN061297	MN065686	MN148124	MN166235	Jian et al. (2020a)
*C.fusiformis*	KUN-HKAS 104514^a^	Yunnan, China 2018	MN061298	MN065687	MN148125	MN166236	Jian et al. (2020a)
*C.fusiformis*	KUN-HKAS 104515^a^	Yunnan, China 2018	MN061300	MN065690	MN148128	MN166239	Jian et al. (2020a)
*C.fusiformis*	KUN-HKAS 104516^a^	Yunnan, China 2018	MN061299	MN065688	MN148126	MN166237	Jian et al. (2020a)
*C.fusiformis*	KUN-HKAS 104517^a^	Yunnan, China 2018	–	MN065689	MN148127	MN166238	Jian et al. (2020a)
*C.giovanellae*	S.F.14368^l^	Trento, Italy 1888	EF413030	EF413027	–	–	Moreno et al. (2007)
*C.giovanellae*	AH 19780^m^	Spain 1998	–	EF413026	–	–	Moreno et al. (2007)
*C.highlandensis*	KUN-HKAS 68389^a^	Yunnan, China 2010	MN061310	MN065700	MN148137	MN166249	Jian et al. (2020a)
*C.highlandensis*	KUN-HKAS 117632^a^	Yunnan, China 2021	ON999061	ON999062	OP006563	OP006564	Jian et al. (2023)
*C.hobsonii*	K(M) 167650^f^	UK 2010	MN855371	–	MN856169	–	Consiglio and Setti (2019)
*C.hobsonii*	K(M) 122842^f^	UK 2004	NR_182819	–	MN856170	–	Consiglio and Setti (2019)
*C.hobsonii*	K(M) 199928^f^	UK 2015	MN855373	–	MN856171	–	Consiglio and Setti (2019)
“*C.hobsonii*”	QYL10	–	OK652826	OK655769	MN092372	MN092373	Peng et al. (2021)
“*C.hobsonii*”	DLL 9779	Queensland, Australia 2010	–	–	KC816916	KC816831	Kluting et al. (2014)
“*C.hobsonii*”	5967 TJB^d^	New York, USA 1988	–	–	KC816917	–	Kluting et al. (2014)
“*C.hobsonii*”	DLL 9586	Queensland, Australia 2009	–	KJ021698	KC816912	KC816828	Kluting et al. (2014)
“*C.hobsonii*”	DLL 9635	Queensland, Australia 2009	–	–	KC816913	KC816829	Kluting et al. (2014)
“*C.hobsonii*”	DLL 9643	Queensland, Australia 2009	–	–	KC816914	–	Kluting et al. (2014)
“*C.hobsonii*”	DLL 9746	Queensland, Australia 2010	–	–	KC816915	KC816830	Kluting et al. (2014)
“*C.hobsonii* grp.”	7051 TJB^d^	North Carolina, USA 1993	–	–	KC816918	–	Kluting et al. (2014)
C.aff.hobsonii	K:M195388^f^	UK 2014	MN855375	–	MN856172	MN856179	Consiglio and Setti (2019)
“C.aff.hobsonii”	UC 1860830^n^	California, USA 2011	–	–	KC816928	KC816841	Kluting et al. (2014)
C.cf.kamaka	KA12-0364°	South Korea 2012	KR673433	–	–	–	Kim et al. (2015)
*C.kamaka*	PDD 96106^p^	New Zealand 2010	NR_137867	–	–	–	Cooper (2014)
*C.lampangensis*	SDBR-CMUJK 0147^q^	Lampang, Thailand 2018	NR_175631	MK764935	MK784129	–	Kumla et al. (2019)
*C.lampangensis*	SDBR-CMUNK 0047^q^	Lampang, Thailand 2018	MK764934	MK773856	MK784128	–	Kumla et al. (2019)
*C.orientalis*	CAL 1613^c^	Kerala State, India 2011	MG345134	MG321558	MG321559	–	Raj and Manimohan (2018)
***C.parasiticus***	**KUN-HKAS 145335^a^**	**Jiangsu, China 2023**	**PQ793168**	**PQ781612**	**PQ788397**	**PQ788404**	**This study**
***C.parasiticus* (H)**	**KUN-HKAS 145336^a^**	**Jiangsu, China 2024**	**PQ793169**	**PQ781613**	**PQ788398**	–	**This study**
***C.parasiticus***	**KUN-HKAS 145337^a^**	**Jiangsu, China 2024**	**PQ793170**	**PQ781614**	**PQ788399**	**PQ788405**	**This study**
*C.passeckerianus*	CBS:299.35^r^	Austria –	MH855682	MH867198	–	–	Vu et al. (2019)
*C.passeckerianus*	P73	South Korea 2015	KY962489	KY963073	–	–	Direct submission
*C.passeckerianus*	P78	South Korea 2015	KY962494	KY963078	–	–	Direct submission
*C.passeckerianus*	K:M134571^f^	UK 2005	MN855376	–	MN856173	–	Consiglio and Setti (2019)
*C.paxilloides*	5809 TJB^d^	California, USA 1987	–	–	KC816919	KC816832	Kluting et al. (2014)
“*C.peri*”	10040 TJB^d^	Chiang Mai, Thailand 2006	–	–	KC816921	KC816834	Kluting et al. (2014)
“*C.peri*”	10033 TJB^d^	Chiang Mai, Thailand 2006	–	–	KC816920	KC816833	Kluting et al. (2014)
“*C.peri*”	10041 TJB^d^	Chiang Mai, Thailand 2006	–	–	KC816922	KC816835	Kluting et al. (2014)
*C.pinsitus*	CBS 623.70^r^	England, UK –	MH859879	MH871665	–	–	Vu et al. (2019)
*C.pinsitus*	G. Immerzeel 1990-11	Netherlands 1990	–	GQ289148	–	–	Co-David et al. (2009)
“*C.prunulus*”	CORT:11CA012^d^	California, USA 2011	–	–	KC816926	KC816839	Kluting et al. (2014)
*C.prunulus*	REH8456^d^	Novgorod Region, Russa 2003	–	–	KC816923	KC816836	Kluting et al. (2014)
“*C.prunulus*”	6805 TJB^d^	New York, USA 1992	–	–	KC816924	KC816837	Kluting et al. (2014)
“*C.prunulus*”	TJB 9425^d^	Dominican Republic 2002	–	–	MN893320	MN893330	Baroni et al. (2020)
“*C.prunulus*”	AFTOL522, TJB6838^d^	USA –	DQ202272	AY700181	–	–	Direct submission
“*C.prunulus*”	TB8229^d^	New York, USA 1996	–	GU384615	GU384650	–	Baroni et al. (2011)
“*C.prunulus*”	TB9663^d^	–	–	GU384614	GU384648	–	Baroni et al. (2011)
*C.prunulus*	KUN-HKAS 96158^a^	Austria 2016	MN061301	MN065691	MN148129	MN166240	Jian et al. (2020a)
*C.prunulus*	KUN-HKAS 123138^a^	France –	OP626992	OP646418	OP939970	OP687894	He et al. (2023)
*C.prunulus*	HMJAU 4521^s^	Kirov, Russia 2006	MN061302	MN065692	MN148117	MN166241	Jian et al. (2020a)
C.cf.prunulus	KUN-HKAS 75845^a^	California, USA 2011	MN061303	MN065693	MN148130	MN166242	Jian et al. (2020a)
*C.ravus*	KUN-HKAS 107043^a^	Yunnan, China 2019	MT345050	MT345055	MT349668	MT349672	Jian et al. (2020b)
*C.reticulosporus*	WU27150^b^	Vienna, Austria 2004	KC885966	HM164412	HM164416	–	Morgado et al. (2016)
*C.rugosiceps*	KUN-HKAS 57003^a^	Yunnan, China 2009	MN061304	MN065694	MN148131	MN166243	Jian et al. (2020a)
*C.rugosiceps*	KUN-HKAS 59455^a^	Yunnan, China 2009	–	MN065696	MN148133	MN166245	Jian et al. (2020a)
*C.rugosiceps*	KUN-HKAS 73232^a^	Yunnan, China 2011	NR_172771	MN065695	MN148132	MN166244	Jian et al. (2020a)
*C.rugosiceps*	KUN-HKAS 107044^a^	Yunnan, China 2019	MT345046	MT345051	–	–	Jian et al. (2020b)
*C.rugosiceps*	KUN-HKAS 115921^a^	Yunnan, China 2017	MZ855871	MZ853557	MZ826364	MZ826362	He and Yang (2022)
*C.scyphoides*	CBS 127.47^r^	France –	MH856181	MH867707	–	–	Vu et al. (2019)
C.cf.scyphoides	KUN-HKAS 104511^a^	Gansu, China 2016	MN061329	MN065720	MN148157	MN166268	Jian et al. (2020a)
*C.sinoapalus*	KUN-HKAS 77037^a^	Jiangxi, China 2012	MN061321	MN065713	MN148149	–	Jian et al. (2020a)
*C.sinoapalus*	KUN-HKAS 82230^a^	Guangzhou, China 2013	MN061320	MN065712	MN148148	–	Jian et al. (2020a)
*C.sinoapalus*	KUN-HKAS 83831^a^	Yunnan, China 2014	–	MN065714	MN148150	–	Jian et al. (2020a)
*C.sinoapalus*	KUN-HKAS 101191^a^	Yunnan, China 2017	NR_172773	MN065711	MN148151	MN166261	Jian et al. (2020a)
*C.sinoapalus*	KUN-HKAS 102737^a^	Yunnan, China 2017	–	MN065709	MN148146	MN166259	Jian et al. (2020a)
*C.sinoapalus*	KUN-HKAS 102807^a^	Yunnan, China 2017	MN061319	MN065710	MN148147	MN166260	Jian et al. (2020a)
*C.subalbidus*	GDGM 72219^t^	Guangdong, China 2018	NR_198267	NG_243733	ON959185	ON959190	Jian et al. (2023)
*C.subalbidus*	GDGM 72229^t^	Guangdong, China 2018	ON963952	ON963946	ON959186	–	Jian et al. (2023)
*C.subscyphoides*	CAL 1325^c^	Kerala State, India 2011	MF927542	MF946580	MF946581	–	Raj and Manimohan (2018)
*C.subscyphoides*	GDGM 72195^t^	Guangdong, China 2018	–	–	ON959188	–	Jian et al. (2023)
*C.subscyphoides*	GDGM 72683^t^	Guangdong, China 2018	ON963953	ON963947	–	–	Jian et al. (2023)
*C.subscyphoides*	GDGM 73056^t^	Guangdong, China 2018	ON963954	ON963948	ON959187	ON959191	Jian et al. (2023)
*C.umbilicatus*	KUN-HKAS 80289^a^	Hunan, China 2013	MN061323	MN065715	MN148152	MN166262	Jian et al. (2020a)
*C.umbilicatus*	KUN-HKAS 80310^a^	Anhui, China 2013	MN061324	MN065716	MN148153	MN166263	Jian et al. (2020a)
*C.umbilicatus*	KUN-HKAS 80370^a^	Fujian, China 2013	MN061325	MN065717	MN148154	MN166264	Jian et al. (2020a)
*C.umbilicatus*	KUN-HKAS 80945^a^	Anhui, China 2013	MN061326	MN065718	MN148155	MN166265	Jian et al. (2020a)
*C.umbilicatus*	KUN-HKAS 104509^a^	Yunnan, China 2017	MN061327	MN065719	MN148156	MN166266	Jian et al. (2020a)
*C.velutinus*	CORT 014618^d^	Dominican Republic 2015	MN784991	–	MN893321	MN893331	Baroni et al. (2020)
*C.venososulcatus*	8111 TJB^d^	Louisiana, USA 1996	–	–	KC816930	–	Kluting et al. (2014)
*C.yunnanensis*	KUN-HKAS 59712^a^	Yunnan, China 2009	MN061307	–	MN148135	–	Jian et al. (2020a)
*C.yunnanensis*	KUN-HKAS 82076^a^	Yunnan, China 2012	MN061306	MN065697	MN148134	MN166246	Jian et al. (2020a)
*C.yunnanensis*	KUN-HKAS 104518^a^	Yunnan, China 2018	MN061308	MN065698	MN148136	MN166247	Jian et al. (2020a)
C.yunnanensis	HMJAU 24677^s^	Sichuan, China 2013	MN061309	MN065699	MN148116	MN166248	Jian et al. (2020a)
“*Clitopilus* sp.”	7130 TJB^d^	New York, USA 1993	–	–	KC816929	–	Kluting et al. (2014)
*Clitopilus* sp.	TB8067^d^	Florida, USA 1996	–	GU384612	GU384649	–	Baroni et al. (2011)
*Clitopilus* sp.	KUN-HKAS 104508^a^	Yunnan, China 2017	MN061311	MN065701	MN148138	MN166250	Jian et al. (2020a)
*Clitopilus* sp.	KUN-HKAS 104512^a^	Yunnan, China 2018	MN061330	MN065721	MN148158	MN166269	Jian et al. (2020a)
*R.alutacea*	5726 TJB^d^	North Carolina, USA 1987	–	–	KC816931	KC816842	Kluting et al. (2014)
*R.asanii*	KATO 3659^u^	Turkey 2015	KX834263	KX834264	–	–	Seslİ and Vizzini (2017)
*R.asanii*	KATO 3657^u^	Turkey 2015	KX834265	–	–	–	Seslİ and Vizzini (2017)
*R.asanii*	NA13102020	East Sussex, UK 2020	MW375030	–	–	–	Aplin et al. (2022)
*R.asyae*	KATO 3640^u^	Trabzon, Turkey 2015	KX834266	KX834267	–	–	Seslİ and Vizzini (2017)
*R.asyae*	KATO 3653^u^	Trabzon, Turkey 2015	KX834268	–	–	–	Seslİ and Vizzini (2017)
*R.asyae*	NA131019^v^	East Sussex, UK 2019	MN840644	–	–	–	Aplin et al. (2022)
*R.aureicystidiata*	PBM 1902^w^	Washington, USA –	–	AY380407	AY337412	–	Matheny (2005)
*R.brunneoaurantiaca*	CAL 1825^c^	West Bengal, India 2019	MW031906	MW031916	–	–	Dutta et al. (2021)
*R.brunneoaurantiaca*	CUH AM720^x^	West Bengal, India 2019	MW023201	MW023223	–	–	Dutta et al. (2021)
*R.brunnescens*	TENN 056140^y^	Tennessee, USA 1985	NR_119914	NG_058820	–	–	Baroni et al. (2011)
*R.brunnescens*	TENN 056140-2^y^	Tennessee, USA 1987	HQ222033	JF706313	–	–	Baroni et al. (2011)
*R.byssisedoides*	AG 2004-04-27	Jena, Germay 2004	–	GQ289212	GQ289279	–	Co-David et al. (2009)
*R.caelata*	511	Germany 2005	–	GQ289208	–	–	Co-David et al. (2009)
“*R.caelata*”	6919 TJB^d^	North Carolina, USA 1992	–	–	KC816933	KC816843	Kluting et al. (2014)
*R.caelata*	J. Parkin^d^	Ontario, Canada 1988	–	–	KC816934	–	Kluting et al. (2014)
*R.caelata*	REH3569^d^	Jurmala, Latvia 1982	–	–	KC816932	–	Kluting et al. (2014)
*R.caelata*	TB5890^d^	–	–	AF261282	–	–	Moncalvo et al. (2002)
“*R.caelata*”	TB6995^d^	–	–	GU384625	GU384652	–	Baroni et al. (2011)
*R.cistetorum*	KATO 4260^u^	Trabzon, Turkey 2019	NR_176724	MT252601	–	–	Seslİ (2021)
*R.collybioides*	10417 TJB^d^	Jujuy, Argentina 2011	–	–	KC816935	KC816844	Kluting et al. (2014)
*R.dominicana*	ANGE 464	Dominican Republic 2014	–	–	MN893322	MN893332	Baroni et al. (2020)
*R.dominicana*	ANGE 473	Dominican Republic 2014	–	–	MN893323	MN893333	Baroni et al. (2020)
*R.formosa*	1061015-6^d^	Catalonia, Spain 2006	KU862856	–	KC816939	KC816849	Kluting et al. (2014)
*R.formosa*	12/198	Latium, Italy 2012	KU862857	–	–	–	Vizzini et al. (2016)
*R.formosa*	12/208	Latium, Italy 2012	KU862858	–	–	–	Vizzini et al. (2016)
*R.formosa*	1071101-4^d^	Catalonia, Spain 2007	KU862860	–	KC816947	KC816857	Kluting et al. (2014)
*R.formosa*	K(M): 158060^f^	England, UK 2006	MZ159381	–	KC816978	KC816885	Direct submission
*R.fuliginea*	E537^d^	Tasmania, Australia 1999	–	–	KC816940	KC816850	Kluting et al. (2014)
*R.fumanellii*	HFRG_PC200928_1	Buckinghamshire, UK 2020	MW401761	–	–	–	Aplin et al. (2022)
*R.fumanellii*	BOLGH_22122001	Tuscany, Italy 2022	OR831361	–	–	–	Direct submission
*R.fumanellii*	MCVE 29550^z^	Veneto, Italy 2017	MH399225	MH399226	–	–	Vizzini et al. (2018)
*R.fusipes*	DLK 587^aa^	Amazonas, Brazil 2012	MN306209	–	–	–	Silva-Filho et al. (2020)
*R.fusipes*	DLK 298^aa^	Amazonas, Brazil 2012	MN306210	–	–	–	Silva-Filho et al. (2020)
*R.gemina*	GZ 2003-09-14	Belgium 2003	–	–	GQ289277	–	Co-David et al. (2009)
“*R.gemina*”	MEN 2001119	– 2001	–	HM164411	–	–	Morgado et al. (2016)
*R.gemina*	CBS 604.76^r^	–	–	AF223168	–	–	Vu et al. (2019)
*R.gemina*	KATO 2658^u^	Turkey 2009	–	KX834269	–	–	Seslİ and Vizzini (2017)
*R.gemina*	CBS 482.50^r^	–	EF421110	AF223167	EF421019	KP255478	Baroni et al. (2011)
*R.griseoaurantia*	CAL 1324^c^	Kerala, India 2011	NR_154435	KX083574	KX083568	–	Hyde et al. (2016)
*R.griseonigrella*	1081204^ab^	Barcelona, Spain 2008	KU862859	–	–	–	Vizzini et al. (2016)
*R.hondensis*	6103 TJB^d^	California, US 1988	–	–	KC816941	KC816851	Kluting et al. (2014)
*R.incarnata*	REH5369	Venezuela 1987	MT254071	–	–	–	Silva-Filho et al. (2020)
*R.indica*	CAL 1323^c^	Kerala, India 2013	KX083569	NG_060166	KX083566	–	Hyde et al. (2016)
*R.lateritia*	Co-David 418	–	–	HM164410	–	–	Morgado et al. (2016)
*R.lateritia*	E1589^d^	Tasmania, Australia 2002	–	–	KC816942	KC816852	Kluting et al. (2014)
*R.luteobrunnea*	CAL 1322^c^	Kerala, India 2010	NR_154434	NG_060167	KX083567	–	Hyde et al. (2016)
*R.luteocinnamomea*	GUA241^d^	Guana Island, UK 1999	–	–	KC816943	KC816853	Kluting et al. (2014)
R.luteocinnamomeavar.fulva	ANGE 169	Dominican Republic 2013	–	–	MN893324	MN893334	Baroni et al. (2020)
*R.matesina*	MCVE 29262^z^	Campania, Italy 2012	KY629961	KY629963	–	–	Crous et al. (2017)
*R.matesina*	MCVE 29261^z^	Campania, Italy 2016	KY629962	KY629964	–	–	Crous et al. (2017)
*R.matesina*	F3-2	Fnaydek, Lebanon 2018	MZ088085	–	–	–	Sleiman et al. (2021)
“*R.mellea*”	ANGE 893	Dominican Republic 2016	MN784993	–	MN893326	–	Baroni et al. (2020)
“*R.mellea*”	TJB 9823^d^	Belize 2004	MN784994	–	–	–	Baroni et al. (2020)
*R.mellea*	NYBG815044	Costa Rica 1986	MN784995	–	–	–	Baroni et al. (2020)
“*R.mellea*”	6883 TJB^d^	Florida, USA 1992	–	MG702608	KC816944	KC816854	Kluting et al. (2014)
“*R.mellea*”	TJB 9805^d^	Dominican Republic 2003	MN784992	–	MN893325	–	Baroni et al. (2020)
R.melleavar.depressa	FW 08/2019	Brazil 2019	MT408926	OL687341	–	–	Xavier et al. (2022)
*R.nuciolens*	WTU-F-074620	Washington, USA 2017	OP828513	–	–	–	Direct submission
*R.nuciolens*	TENN:076696^y^	Washington, USA 2021	ON478246	–	–	–	Direct submission
*R.nuciolens*	iN147673878	California, USA 2023	OR162504	–	–	–	Direct submission
*R.nuciolens*	iN147466901	California, USA 2023	OR168848	–	–	–	Direct submission
*R.pakistanica*	LAH37947^i^	Punjab, Pakistan 2022	OR606543	OR606541	–	–	Khan and Khalid (2024)
*R.pakistanica*	LAH37948^i^	Punjab, Pakistan 2022	OR606544	OR606542	–	–	Khan and Khalid (2024)
*R.pallidogrisea*	CORT 013944^d^	Australia –	NR_154437	–	–	–	Direct submission
*R.pallidogrisea*	118	Tasmania, Australia 2004	–	GQ289216	GQ289283	–	Co-David et al. (2009)
*R.pallidogrisea*	E652^d^	Tasmania, Australia 1999	–	–	KC816968	KC816875	Kluting et al. (2014)
*R.paurii*	JM99/233	Uttaranchal, India 1999	–	AY286004	–	–	Moncalvo et al. (2004)
*R.paurii*	JM99/233-2	Uttaranchal, India 1999	–	–	KC816969	KC816876	Kluting et al. (2014)
*R.praesidentialis*	MCVE 21991^z^	Italy –	EF679793	–	–	–	Consiglio et al. (2007)
*R.pruinosostipitata*	MCA1492	Guyana –	–	GU384627	GU384653	–	Baroni et al. (2011)
*R.pseudoalutacea*	TJB 9466^d^	Dominican Republican 2003	–	–	MN893327	MN893335	Baroni et al. (2020)
*R.pseudoalutacea*	TJB 9507^d^	Dominican Republican 2003	–	–	MN893328	MN893336	Baroni et al. (2020)
*R.pseudopiperita*	E1159^d^	Tasmania, Australia 2001	–	–	KC816979	KC816886	Kluting et al. (2014)
*R.pseudopiperita*	162	Tasmania, Australia 2004	–	GQ289217	GQ289284	–	Co-David et al. (2009)
*R.reticulata*	E2183^d^	Tasmania, Australia 2005	–	–	KC816980	KC816887	Kluting et al. (2014)
*R.rhizogena*	5551 TJB^d^	North Carolina, USA 1987	–	–	KC816981	KC816888	Kluting et al. (2014)
*R.roseiavellanea*	8130 TJB^d^	Louisiana, USA 1996	–	KR869930	KC816982	KC816889	Kluting et al. (2014)
*R.roseiavellanea*	PBM4056	Tennessee, USA –	MF686525	–	–	–	Direct submission
*R.roseiavellanea*	ANGE 947	Dominican Republic 2017	–	–	MN893329	MN893337	Baroni et al. (2020)
*R.rubrobrunnea*	CAL 1387^c^	Kerala, India 2014	KX951452	–	–	–	Crous et al. (2016)
*Rhodocybe* sp.	DLL9851	New South Wales, Australia 2010	–	–	KC816986	KC816893	Kluting et al. (2014)
*Rhodocybe* sp.	DLL9846	New South Wales, Australia 2010	–	–	KC816985	KC816892	Kluting et al. (2014)
*Rhodocybe* sp.	DLL9860	New South Wales, Australia 2010	–	–	KC816987	KC816894	Kluting et al. (2014)
*Rhodocybe* sp.	DLL9952	New South Wales, Australia 2010	–	–	KC816988	KC816895	Kluting et al. (2014)
*Rhodocybe* sp.	DLL9957	New South Wales, Australia 2010	–	–	KC816989	KC816896	Kluting et al. (2014)
*Rhodocybe* sp.	DLL10218	New South Wales, Australia 2011	–	–	KC816990	KC816897	Kluting et al. (2014)
*Rhodocybe* sp.	DLL10032	Queensland, Australia 2011	–	–	KC816991	KC816898	Kluting et al. (2014)
*Rhodocybe* sp.	KUN-HKAS 89081^a^	Yunnan, China 2023	MZ675559	MZ675570	MZ681892	MZ681870	He and Yang (2022)
*Rhodocybe* sp.	MEL:2382939	Palmerston, Australia 2014	KP012803	–	–	–	Direct submission
*Rhodocybe* sp.	MEL:2382705	Australia 2014	KP012885	–	–	–	Direct submission
*Rhodocybe* sp.	KS-RE53	New Zealand –	–	MK277733	–	–	Varga et al. (2019)
*Rhodocybe* sp.	Buyck 99.152	Madagascar –	–	MK278564	–	–	Varga et al. (2019)
*Rhodocybe* sp.	Sulzbacher 340	Brazil –	LT594979	–	–	–	Sulzbacher et al. (2017)
*Rhodocybe* sp.	Sulzbacher 413	Brazil –	LT594984	–	–	–	Sulzbacher et al. (2017)
*Rhodocybe* sp.	HFRG_EJ171117_1	Hampshire, UK 2017	MW397197	MW397521	–	–	Aplin et al. (2022)
*Rhodocybe* sp.	iN130319090	Indiana, USA 2022	OP749482	–	–	–	Direct submission
*Rhodocybe* sp.	iN129753148	Indiana, USA 2022	OP749140	–	–	–	Direct submission
*Rhodocybe* sp.	iN130020200	Indiana, USA 2022	OP643320	–	–	–	Direct submission
*Rhodocybe* sp.	AD5 (TENN)^y^	Tennessee, USA 2011	MF773647	–	–	–	Direct submission
*R.stipitata*	5523 TJB^d^	Tennessee, USA 1987	–	–	KC816993	–	Kluting et al. (2014)
*R.spongiosa*	MCA2129		–	GU384628	GU384657	–	Baroni et al. (2011)
*R.subasyae*	HMJAU56921-1^s^	Jilin, China 2020	MW298803	–	–	–	Sun and Bau (2023)
*R.subasyae*	HMJAU56921-2^s^	Jilin, China 2020	MW298804	–	–	–	Sun and Bau (2023)
*R.subasyae*	HMJAU56921-3^s^	Jilin, China 2020	MW298805	–	–	–	Sun and Bau (2023)
*R.tugrulii*	KATO 3340^u^	Trabzon, Turkey 2014	KX271751	KX271754	–	–	Vizzini et al. (2016)
*R.tugrulii*	MSNG3938	Italy –	KY945354	–	–	–	Direct submission
*R.tugrulii*	CORT:14755^d^	New York, USA 2018	MZ322093	–	–	–	Direct submission
*R.tugrulii*	IMG-7316^d^	New York, USA 2017	MG050105	MG050111	–	–	Direct submission
*R.tugrulii*	WU-MYC 0010084^b^	Burgenland, Austria 1991	OP363995	–	–	–	Vizzini et al. (2023)
*R.tugrulii*	WU-MYC 0022202^b^	Niederosterreich, Austria 2002	OP363994	OP363999	OP381082	OP381084	Vizzini et al. (2023)
*R.tugrulii*	WU-MYC 0006178^b^	Niederosterreich, Austria 1987	–	OP364000	–	–	Vizzini et al. (2023)
*R.tugrulii*	WU-MYC 0006320^b^	Niederosterreich, Austria 1987	OP363992	OP363997	OP381080	OP381083	Vizzini et al. (2023)
*R.tugrulii*	WU-MYC 0004222^b^	Niederosterreich, Austria 1984	OP363991	–	–	–	Vizzini et al. (2023)
*R.tugrulii*	WU-MYC 0003753^b^	Niederosterreich, Austria 1984	OP363993	OP363998	OP381081	–	Vizzini et al. (2023)
*R.tugrulii*	GB-013 1395	Skaane, Sweden 1983	OP363996	OP364001	–	–	Vizzini et al. (2023)
***R.zijinshanensis* (H)**	**KUN-HKAS 145338^a^**	**Jiangsu, China 2024**	**PQ793171**	**PQ781615**	**PQ788400**	**PQ788406**	**This study**
***R.zijinshanensis***	**KUN-HKAS 145339^a^**	**Jiangsu, China 2024**	**PQ793172**	**PQ781616**	**PQ788401**	**PQ788407**	**This study**
*Lulesiaumbrinomarginata*	MHHNU 20023-2	Guangdong, China 2023	PP060632	PP059607	PP158704	PP158696	Xiao et al. (2024)
*Lulesiaorientalis*	KUN-HKAS 75548^a^	Hubei, China 2012	MN061333	MN065727	MN148164	MN166275	Jian et al. (2020a)
*Clitopilopsisalbida*	KUN-HKAS 104520^a^	Yunnan, China 2018	MN061336	MN065731	MN148168	MN166279	Jian et al. (2020a)
*Clitopilopsishirneola*	MEN 199956	Italy –	KC710132	GQ289211	GQ289278	–	Co-David et al. (2009)

^a^ The Cryptogamic Herbarium of Kunming Institute of Botany, the Chinese Academy of Sciences, Kunming, China (KUN-HKAS) ^b^Herbarium, Institute of Botany, University of Vienna, Austria (WU) ^c^Central National Herbarium, Kolkata, India (CAL) ^d^State University of New York College at Cortland Herbarium, Cortland, New York, USA (CORT) ^e^ The Leiden Herbarium of Naturalis Biodiversity Center, Leiden, Netherland (L) ^f^ The Royal Herbarium, Royal Botanic Garden, Kew, Richmond, Surrey, England, UK (KEW) ^g^Associazione Micologica Bresadola, Trento, Italy (AMB) ^h^The Herbarium of Mycology, Jilin Agricultural University, Changchun, Jilin, China (HMJAU) ^i^The Lahore, Institute of Botany, University of the Punjab, Pakistan (LAH) ^j^Herbarium generale del Dipartimento di Biologia Vegetale, Università degli Studi di Torino, Italy (TO) ^k^ The Herbarium of Soil and Fertilizer Institute, Sichuan Academy of Agricultural Sciences, Sichuan, China (SAAS) ^l^ The Swedish Museum of Natural History, Stockholm, Sweden (S) ^m^The Herbarium of the University of Alcalá, Madrid, Spain (AH) ^n^Jepson Herbarium, University of California, Berkeley, California, USA (JEPS) ° The Herbarium of Korea National Arboretum, Gyeonggi-do, Republic of Korea (KH) ^p^ New Zealand Fungal Herbarium, Auckland, New Zealand (PDD) ^q^The Herbarium of the Sustainable Development of Biological Resources Laboratory, Chiang Mai University, Thailand (SDBR-CMU) ^r^Centraalbureau voor Schimmelcultures, Utrecht, Netherland (CBS) ^s^ The Herbarium of Mycology, Jilin Agricultural University, Changchun, Jilin, China (HMJAU) ^t^The Mycological Herbarium of the Guangdong Institute of Microbiology, Guangdong Academy of Sciences, Guangzhou, China (GDGM) ^u^ Karadeniz Technical University Faculty of Forestry Herbarium, Trabzon, Turkey (KATO) ^v^ United States National Arboretum, USDA-ARS, Washington, USA (NA) ^w^ The Herbarium of Mahidol University, Nakhon Pathom, Thailand (PBM) ^x^The Calcutta University Herbarium, Kolkata, West Bengal, India (CUH) ^y^The Herbarium of University of Tennessee – Knoxville, Tennessee, USA (TENN) ^z^New Herbarium of Museo di Storia Naturale di Venezia, Venezia, Italy (MCVE) ^aa^Herbário of Instituto Nacional de Pesquisas da Amazônia, Amazonas, Brazil (INPA) ^ab^Herbier of Université de Lille, Lille, France (LIP) (Newly-generated information of specimens and sequences are in bold).

For the sequence alignments, Sequencher 4.1.4 (Gene Code Corp., Ann Arbor, MI) was used to concatenate sequences obtained from both direction (5’–3’ & 3’–5’), to remove regions with heavy peaks and to merge degenerate bases. The sequences were then aligned using MAFFT 7.526 (Katoh et al. 2005) and manually checked in BioEdit 7.7.1 (Hall 1999). Separate single-gene analyses were performed to exclude conflicts amongst topologies using Maximum Likelihood and Bayesian Inference. Subsequently, Phyutility 2.2 (Smith and Dunn 2008) was employed to combine all the separate single-gene datasets. Any deficiencies in the DNA fragment sequences were treated as missing data in the subsequent analyses. A super-matrix was generated by combining sequences of all four loci.

Under the Akaike Information Criterion (AIC), the best-fitted substitution model for each dataset was determined with MrModelTest 2.3 (Nylander 2004). Phylogenetic analyses were conducted using Maximum Likelihood (ML) with RAxML 7.2.6 (Stamatakis 2006) and Bayesian Inference (BI) with MrBayes 3.2.6 (Ronquist and Huelsenbeck 2003). In view of the close kinship amongst *Lulesia* Singer & *Clitopilus*, as well as *Clitopilopsis* Maire & *Rhodocybe*, *Lulesiaumbrinomarginata* Y.Q. Xiao et al. and *Lulesiaorientalis* (S.P. Jian & Zhu L. Yang) Vizzini et al. were selected as outgroups for the phylogenetic tree of *Clitopilus*. Similarly, *Clitopilopsisalbida* S.P. Jian & Zhu L. Yang and *Clitopilopsishirneola* (Fr.) Kühner were chosen as outgroups for the phylogenetic tree of *Rhodocybe*.

For ML analyses, the GTRGAMMAI model was applied to the combined dataset, with statistical support for internodes obtained through non-parametric bootstrapping with 1000 replications. For the BI analyses of the combined dataset, a partitioned mixed model was implemented, defining the sequences of ITS, LSU, *RPB2* and *TEF1* as four independent partitions, with each gene estimated using different model parameters. The best-selected model was employed and the Markov Chain Monte Carlo (MCMC) chain was run for four million generations. The STOPRULE command was set with STOPVAL = 0.01 and trees were sampled every 100 generations. We verified chain convergence using Tracer 1.5 (http://tree.bio.ed.ac.uk/software/tracer) to ensure sufficiently large effective sample size (ESS) values greater than 200. The combined tree was summarised using the sump and sumt commands with a 25% burn-in.

## ﻿Results

### ﻿Phylogenetic analyses

**Figure 1.**
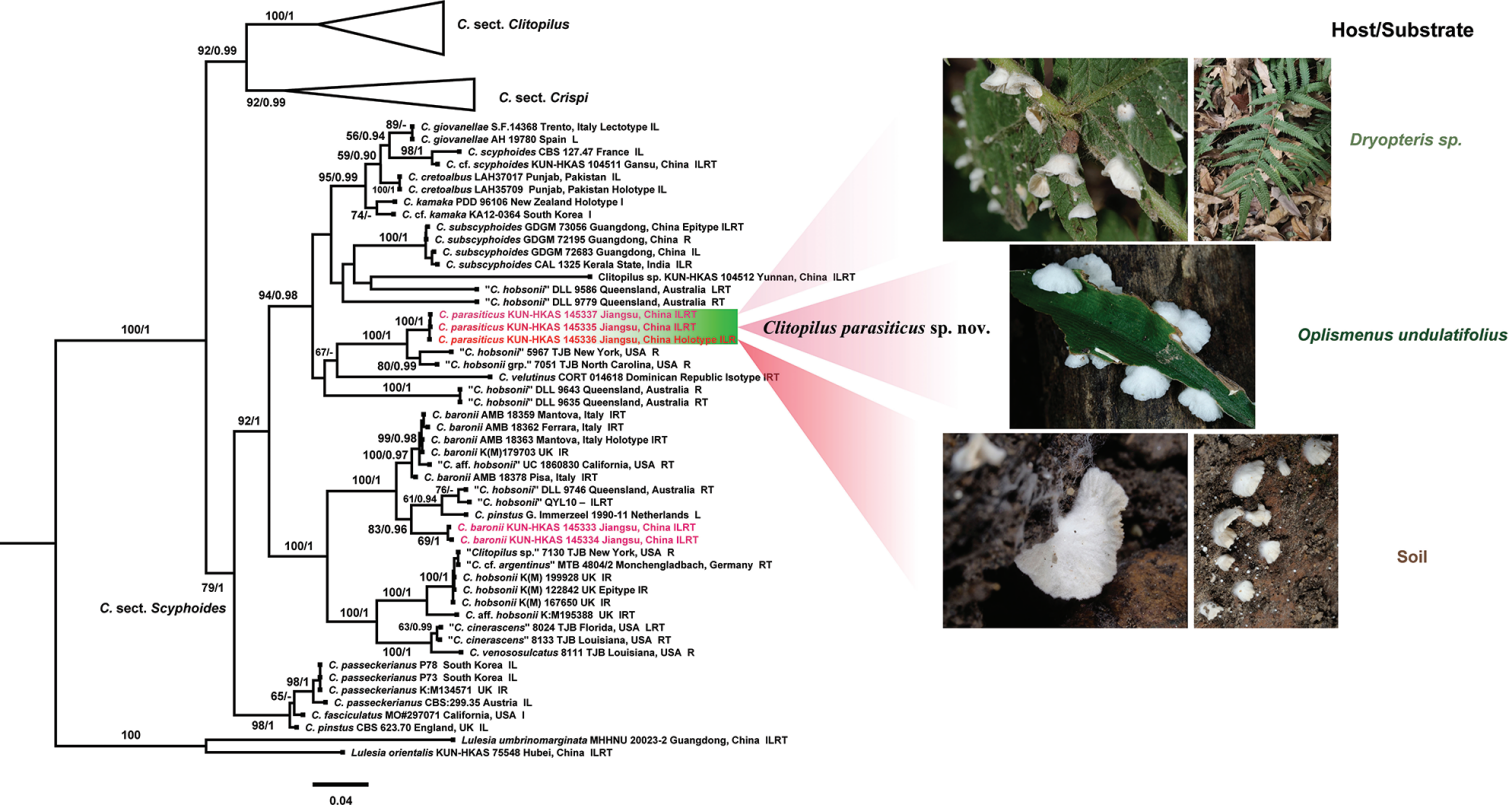
Phylogenetic relationships amongst representative species of *Clitopilus* were inferred from a multigene dataset (ITS-LSU-*RPB2*-*TEF1*) using both ML and BI methods (only shown the ML tree). Supported branches indicate bootstrap supports (BS > 50%) and posterior probabilities (PP > 0.90). Sequences from type specimens (holotype, epitype or isotype) are marked, while new and new record taxa are highlighted in red. The abbreviations ILRT stand for: I = ITS, L = LSU, R = *RPB2* and T = *TEF1*.

**Figure 2.**
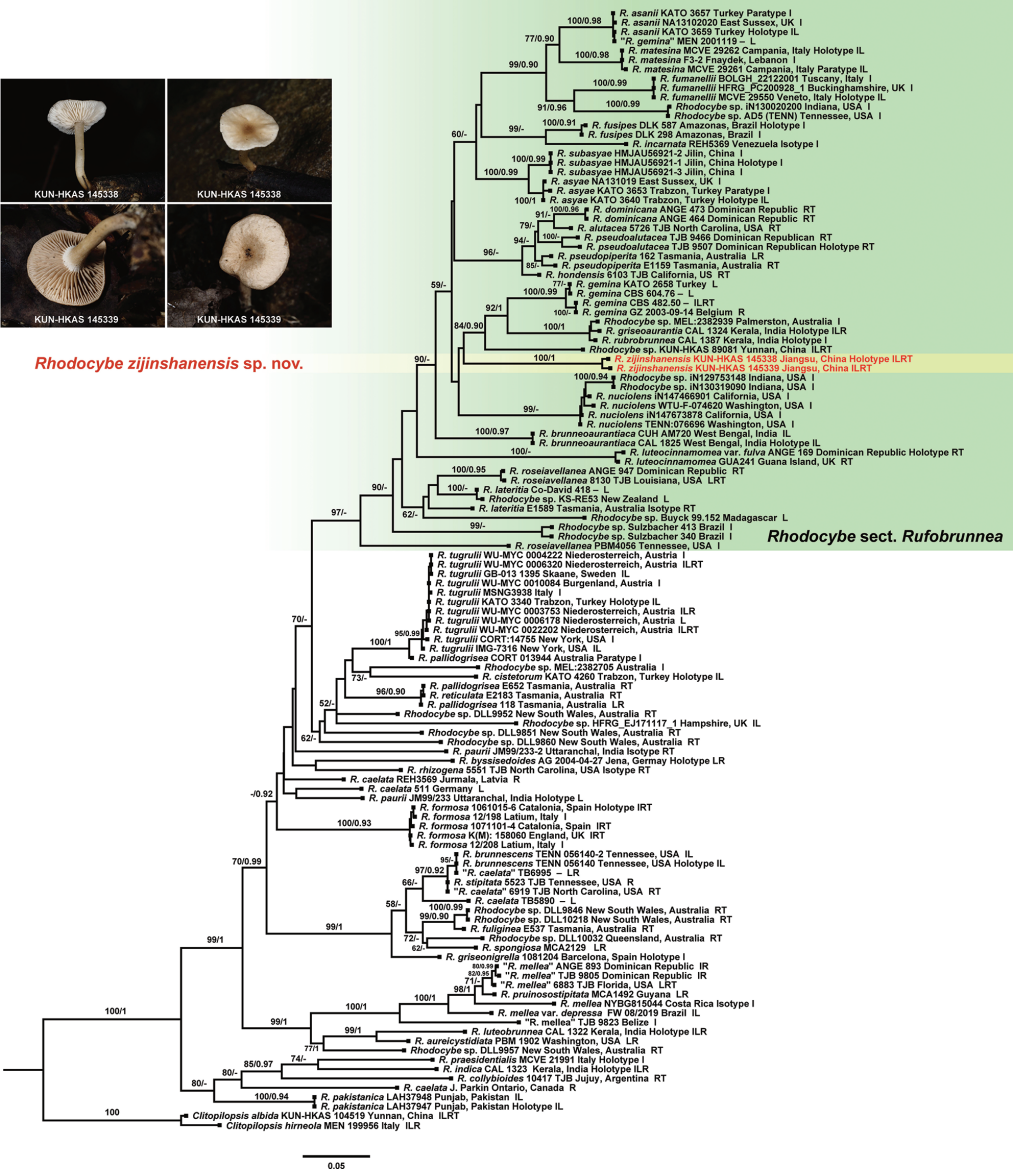
Phylogenetic relationships amongst representative species of *Rhodocybe* were inferred from a multigene dataset (ITS-LSU-*RPB2*-*TEF1*) using both ML and BI methods (only shown the ML tree). Supported branches indicate bootstrap supports (BS > 50%) and posterior probabilities (PP > 0.90). Sequences from type specimens (holotype, paratype or isotype) are marked, while new and new record taxa are highlighted in red. The abbreviations ILRT stand for: I = ITS, L = LSU, R = *RPB2* and T = *TEF1*.

No topological inconsistency was detected between the ML and BI analyses, both for the individual genes and the multigene data. The phylogenetic tree inferred from the ML strategy is presented, with statistical results from both ML (Bootstrap Supports, BS) and BI (Posterior Probabilities, PP) displayed on the branches (see Figs 1, 2). The best-fit model for ML and BI analyses was the GTR+I+R model. In the multigene matrix for *Clitopilus*, we assembled a total of 131 sequences from four genes: 96 for ITS, 89 for LSU, 110 for *RPB2* and 78 for *TEF1*. Analogously, the multigene matrix for *Rhodocybe* included 120 sequences derived from the same four genes, with 72 for ITS, 48 for LSU, 55 for *RPB2* and 37 for *TEF1*. Finally, the dataset for *Clitopilus* included 3664 sites in total, with 788 from ITS, 957 from LSU, 784 from *RPB2* and 1135 from *TEF1*. Similarly, the dataset for *Rhodocybe* included 3881 sites, with 960 from ITS, 985 from LSU, 799 from *RPB2* and 1137 from *TEF1*.

In the phylogenetic tree of *Clitopilus* (Fig. 1), species with parasitic abilities are clustered together in Clitopilussect.Scyphoides Singer, representing a new species. The species collected from rotten wood are positioned close to *C.baronii* Consiglio & Setti. Furthermore, the phylogenetic tree of *Rhodocybe* (Fig. 2) shows that other species collected from rotten wood are clustered within Rhodocybesect.Rufobrunnea Singer ex T.J. Baroni, also signifying a new species.

### ﻿Morphological observations and SEM

**Figure 3.**
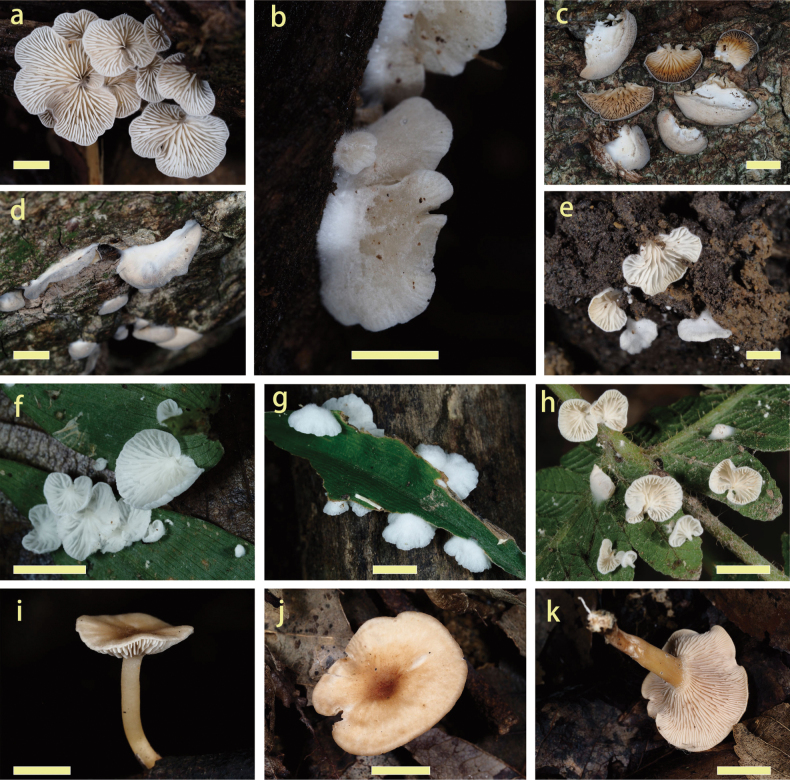
Basidiomata of *Clitopilus* and *Rhodocybe***a–d***Clitopilusbaronii* (**a, b**KUN-HKAS 145333; **c, d**KUN-HKAS 145334) **e–h***Clitopilusparasiticus* (**e**KUN-HKAS 145336, holotype; **f, g**KUN-HKAS 145335; **h**KUN-HKAS 145337) **i–k***Rhodocybezijinshanensis* (KUN-HKAS 145338, holotype). Scale bar: 5 mm.

**Figure 4.**
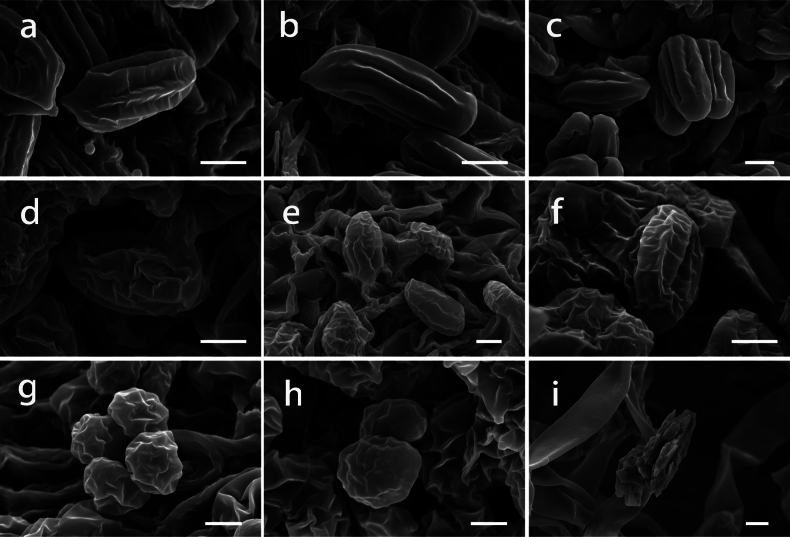
Basidiospores and crystals of *Clitopilus* and *Rhodocybe* reveal by SEM**a–c***Clitopilusbaronii* (KUN-HKAS 145334) **d–f***Clitopilusparasiticus* (KUN-HKAS 145336, holotype) **g, h***Rhodocybezijinshanensis* (KUN-HKAS 145338, holotype) **i** the crystals around the hyphae in pileipellis of *Clitopilusparasiticus* (KUN-HKAS 145336, holotype). Scale bar: 2 μm.

The images of fresh basidiomata, substrate and habitats of the collected specimens are shown in Fig. 3. Scanning Electron Microscopy revealed that the ornamentation of basidiospores provides some extra valuable information (Fig. 4). The basidiospores of *Clitopilus* exhibit several classical longitudinal ridges (Fig. 4a–f), while those of *Rhodocybe* are characterised by undulate-pustulate walls (Fig. 4g–h). In addition, crystals on the pileus hyphae of the new species in *Clitopilus* was also identified (Fig. 4i).

### ﻿Taxonomy

#### ﻿ Clitopilus parasiticus

Taxon classificationFungiAgaricalesEntolomataceae

S.P. Jian, X. Chen & Z.H. Zhang sp. nov.

AB003066-67D0-5C2A-B052-B024B0DE1F9C

857348

**Figure 5.**
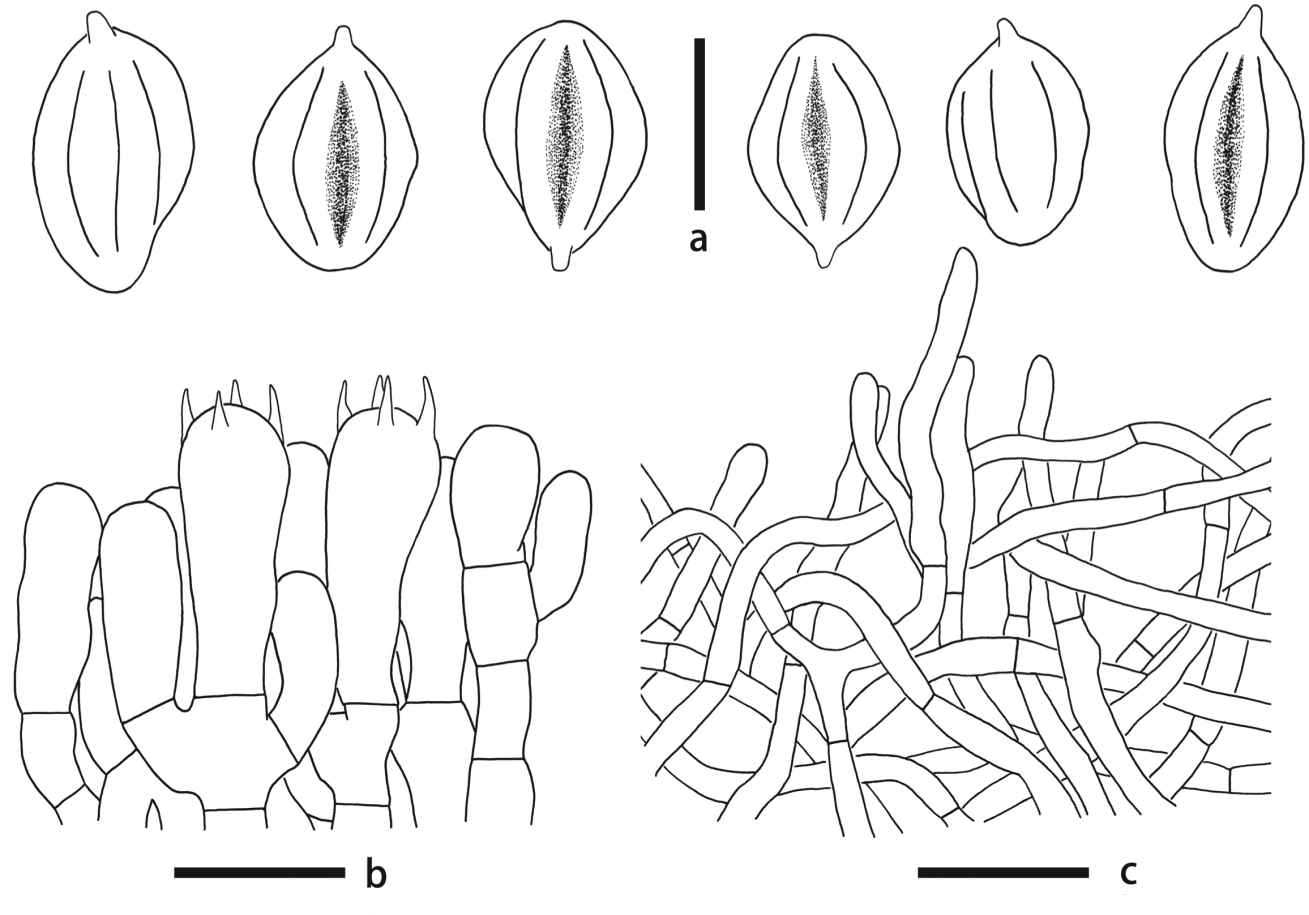
Microscopic features of *Clitopilusparasiticus* (KUN-HKAS 145336, holotype) **a** basidiospores **b** hymenium and subhymenium **c** pileipellis. Scale bars: 5 μm (**a**); 10 μm (**b**); 20 μm (**c**).

Figs 1 , 3e–h , 4d–f , 5a–c

##### Holotype.

China • Jiangsu Province, Nanjing City, Zijinshan, E 118.83, N 32.08, alt. 32 m, scattered on soil, in the mixed broadleaf (i.e. *Quercusvariabilis*, *Robiniapseudoacacia*, *Osmanthusfragrans*, *Broussonetiapapyrifera*, *Ilexlatifolia* and *Yulania* sp.) forest, 15 August 2024, collected by X. Chen and Z.H. Zhang, CX 966 (KUN-HKAS 145336). GenBank: ITS = PQ793169; LSU = PQ781613; *RPB2* = PQ788398.

##### Etymology.

“*parasiticus*” is proposed by its biotrophic behaviour.

##### Diagnosis.

*Clitopilusparasiticus* is similar to *C.hobsonii*, but differs by the tomentose pileus, explanate margin and smaller basidiospores.

##### Description.

Basidiomata pleurotoid to conchoid, small size. Pileus 2–8 mm, convex; surface whitish (#b4c4cb) to chalk white (#e3edf3), with fine tomentose texture usually extending beyond the margin and densely woolly-tomentose at the base; margin typically applanate; context less than 1 mm thick. Lamellae meeting at an excentric point, whitish (#c6d4d3) to yellowish-white (#d3dad4) to yellowish (#dac7ac), slightly dense or crowded, edges entire and concolorous, lamellulae numerous. Stipe absent or very short, eccentric to lateral, measuring 1–2 × 0.2–0.5 mm, concolorous with lamellae. The base with white (#dddddf) mycelium. Odour none.

Basidiospores (5) 5.5–8.5 × 3.5–5.0 (5.5) μm, L_m_ × W_m_ = 6.6 (± 0.63) × 4.2 (± 0.34) μm, Q = 1.20–1.90 (Q_avg_ = 1.55 ± 0.13) [186/9/3], hyaline, ellipsoid to broadly fusiform, subovoid in profile and face view, slightly angled in polar view, with 7–9 inconspicuous or obscure longitudinal ridges in total. Basidia 16–23 × 6–9.5 μm, clavate, hyaline, 4-spored, rarely 2-spored; sterigmata 2–3 μm. Lamellar trama subregular, composed of thin-walled, hyaline, cylindrical hyphae with a diameter of 2.5–9 μm. Lamellae edges fertile. Pleurocystidia and cheilocystidia absent. Pileipellis a cutis composed of sparsely arranged, thin-walled, hyaline, smooth, interwoven, cylindrical hyphae with a diameter of 3–5 μm, sometimes featuring erect hyphae; crystals present around the hyphae, square to subsquare, measuring 3 × 3 μm to 14 × 15 μm in area; pileal trama subregular, composed of hyaline, filamentous, thin-walled hyphae, with a diameter of 3–7.5 μm. Clamp connections absent.

##### Ecology and distribution.

Solitary, scattered on soil, lignicolous or gregariously living on leaves of plants (*Dryopteris* sp. and *Oplismenusundulatifolius*) in the mixed broadleaf forest, distributed in Jiangsu Province, China, in August.

##### Additional specimens examined.

China • Jiangsu Province, Nanjing City, Zijinshan, alt. 48 m, dispersedly or gregariously lignicolous or living on twigs or leaves of *Oplismenusundulatifolius*, in the mixed broadleaf (i.e. *Quercusvariabilis*, *Quercusaliena*, *Cunninghamialanceolata*, *Symplocostanakana*, *Celtissinensis* and *Ilexcornuta*) forest, 16 August 2023, collected by X. Chen and Z.H. Zhang, CX 628 (KUN-HKAS 145335); same places, alt. 48 m, dispersedly or gregariously living on leaves of *Dryopteris* sp., 16 August 2024, collected by X. Chen and Z.H. Zhang, CX 967 (KUN-HKAS 145337).

##### Notes.

*Clitopilusparasiticus* belongs to Clitopilussect.Scyphoides (Fig. 1). This new taxon is similar to *C.hobsonii*, *C.daamsii*, *C.passeckerianus*, *C.pinsitus* and *C.baronii*. *Clitopilushobsonii* was originally described from Britain and exhibits both saprophytic and parasitic abilities. It resembles *C.parasiticus* in its living habits and the shape of its basidiomata, but differs from the latter by its involute or inflexed margins of the pileus and larger basidiospores (L_m_ × W_m_ = 7.5 × 5 μm) (Orton 1960; Noordeloos 1984; Noordeloos 1988). Meanwhile, *C.daamsii* was also similar to *C.parasiticus* in outline; however, it differs due to its xylogenous or mycoparasitic behaviour, involute margin of pileus and larger basidiospores (8–11.5 × 4.8–6.6 μm) (Noordeloos 1984). Another closely-related species is *C.passeckerianus*, which has sessile basidiomata and a white pileus. However, the habit of growing on mushroom-beds, basidiomata size (8–40 mm), the reniform to spathulate shape of the pileus and larger basidiospores (7–9 × 4–5 μm) of *C.passeckerianus* significantly differs from *C.parasiticus* (Pilát 1935; Noordeloos 1993). *Clitopiluspinsitus* was first collected from Sweden and was found growing on the trunk of *Quercus*. This species is characterised by its spatulate, white pileus (15–40 mm) and ellipsoidal to amygdaliform basidiospores (7.5–9 × 4.6–5.3 μm) with 7–8 obscure longitudinal ridges (Josserand 1937; Singer 1946a). Lastly, *C.baronii*, recently described by Consiglio and Setti (2019) in Marmirolo, Italy, resembles *C.parasiticus*, but can be differentiated by its larger pileus (5–40 mm) and basidiospores (L_m_ × W_m_ = 7.6 × 5.0 μm), as well as its lageniform cheilocystidia.

#### ﻿ Clitopilus baronii

Taxon classificationFungiAgaricalesEntolomataceae

Consiglio & Setti, Index Fungorum 427: 1. 2019.

2AB350EB-E7CE-5BC5-99AB-3D748AC95A56

**Figure 6.**
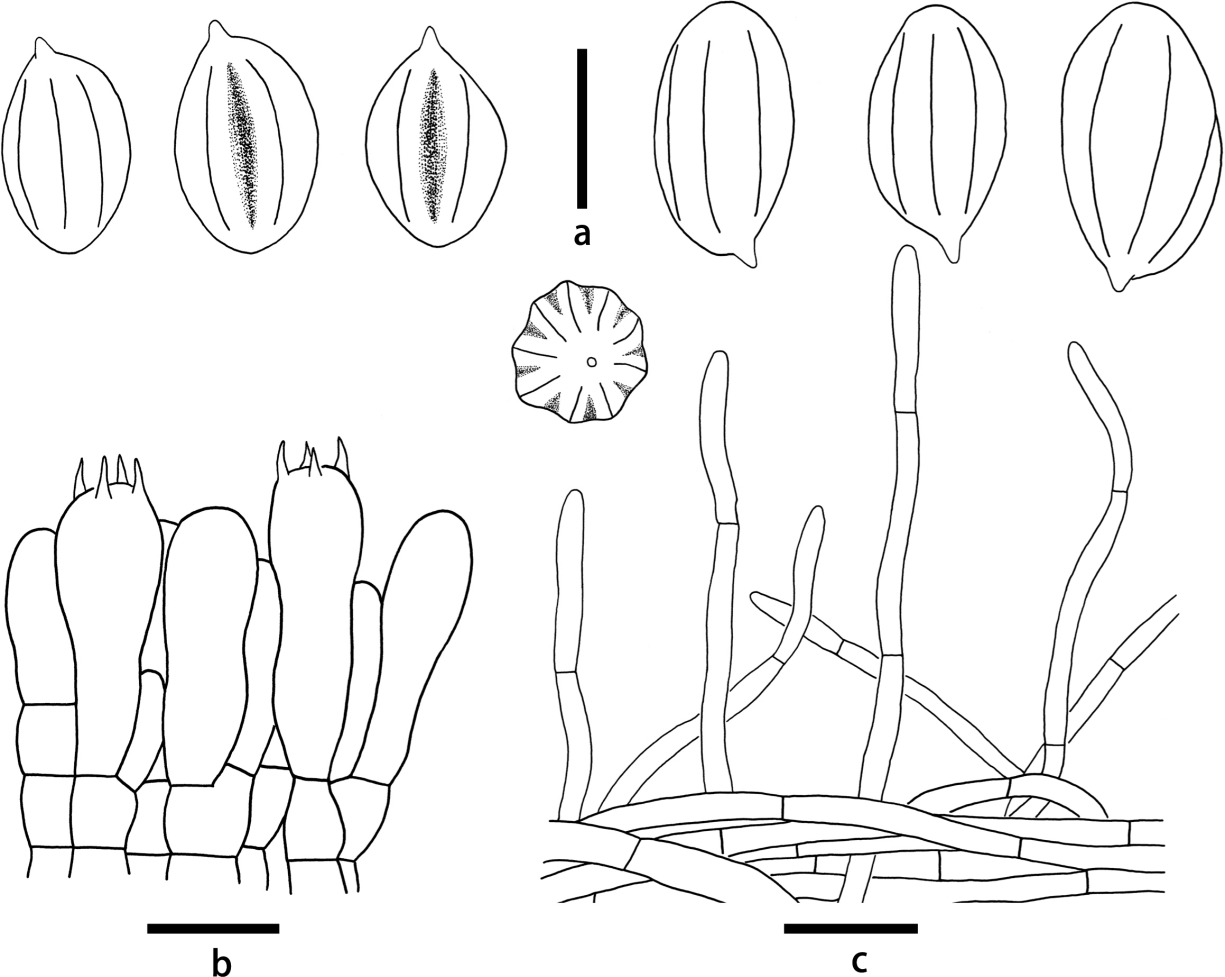
Microscopic features of *Clitopilusbaronii* (KUN-HKAS 145334) **a** hymenium and subhymenium **b** basidiospores **c** pileipellis. Scale bars: 5 μm (**a**); 10 μm (**b**); 20 μm (**c**).

Figs 3a–d , 4a–c , 6a–c

##### Description.

Basidiomata pleurotoid to crepidotoid, small size. Pileus 3–15 mm wide, convex then expanded; surface yellowish-white (#9a8a7a), greyish (#a6a39f) to bluish-grey (#6a757b), usually subtly woolly-tomentose at the base then reduced to border; margin slightly incurved, even, sometimes faintly striated; context less than 1 mm thick. Lamellae whitish (#a9a7a8) to yellowish (#9d896d), sometimes hygrophanous, slightly dense or crowded, edges entire and concolorous, lamellulae numerous. Stipe absent; the base with white (#e9ebed) mycelium. Odour none.

Basidiospores (6) 6.5–9.5 (11) × 4–5 (5.5) μm, L_m_ × W_m_ = 7.5 (± 1.01) × 4.5 (± 0.35) μm, Q = 1.4–1.98 (Q_avg_ = 1.66 ± 0.14) [43/2/2], hyaline, ellipsoid to fusiform, subovoid in profile and face view, slightly angled in polar view with 8–10 inconspicuous or obscure longitudinal ridges in total. Basidia 17.5–24 × 6.5–9 μm, clavate, hyaline, 2- or 4-spored; sterigmata 3–5.5 μm. Lamellar trama subregular, composed of thin-walled, hyaline, cylindrical hyphae with a diameter of 2.5–9 μm. Lamellae edges fertile. Pleurocystidia and cheilocystidia absent, but occasionally forming a few cylindrical tramal hyphae with a diameter of 2–3 μm over the edge. Pileus context about 150–200 μm thick. Pileipellis a cutis composed of compactly arranged, thin-walled, hyaline, smooth, cylindrical hyphae with a diameter of 3.5–9 μm, featuring sparely arranged and erect hyphae with a diameter of 2–3 μm; pileal trama subregular or irregular, composed of hyaline, filamentous, thin-walled hyphae, with a diameter of 2.5–8.5 μm. Clamp connections absent.

##### Ecology and distribution.

Lignicolous, scattered or gregarious on rotten wood in the mixed broadleaf forest, distributed in Jiangsu Province, China, in May.

##### Additional specimens examined.

China • Jiangsu Province, Nanjing City, Zijinshan, alt. 42 m, scattered or gregarious on rotten wood (*Quercus* sp.), in the mixed broadleaf (i.e. *Quercusacutissima*, *Quercusaliena*, *Celtissinensis*, *Liquidambarformosana* and *Cunninghamialanceolata*) forest, 7 May 2023, collected by X. Chen, CX 119 (KUN-HKAS 145333); same places, alt. 38 m, scattered on rotten wood (*Quercus* sp.), in the mixed broadleaf (*Quercusglauca*, *Pterocaryastenoptera*, *Ilexchinensis*, *Cunninghamialanceolata*, *Ilexcornuta*, *Liquidambarformosana* and *Ligustrumlucidum*) forest, 9 May 2023, collected by X. Chen, CX 134 (KUN-HKAS 145334).

##### Notes.

*Clitopilusbaronii* belongs to C.sect.Scyphoides (Fig. 1). In the original description, this species was found growing on a decaying trunk of *Quercus* sp. It is characterised by its sessile basidiomata, orbicular to conchate white pileus, cream-rose lamellae, ellipsoidal to subamygdaliform basidiospores with 8–10 obscure longitudinal ridges and lageniform cheilocystidia. The macro- and microscopic features of our specimens (KUN-HKAS 145333 & 145334) closely match those described in the primary literature (Consiglio and Setti 2019). However, we did not observe any lageniform cheilocystidia in our specimens; we only identified a few thin cylindrical tramal hyphae over the edge. This observation aligns with findings by Noordeloos (1984) regarding *C.daamsii*, particularly in some older specimens. In our previous study, we also noted this phenomenon of thin cylindrical tramal hyphae at the edge in *C.crispus* Pat. However, this occurrence was generally casual and rare.

In the phylogenetic tree of *Clitopilus*, we could discover some unusual results regarding *C.baronii*. In the combined multigene analyses (ITS-LSU-*RPB2*-*TEF1*), our specimens were found to separate from the clades of *C.baronii* and grouped (BS/PP = 69/1) closer to *C.pinstus* (G. Immerzeel 1990-11). When we compared the different genes separately between our samples and holotype of *C.baronii* (AMB 18363), we found that our samples exhibited over 99% similarity in ITS region. However, the similarity was only about 90% for both *RPB2* and *TEF1*. For ITS, we have tested them several times in different companies, but all yielded consistent results. Regarding *RPB2* and *TEF1*, we did not detect any issues with the original data; all sequences were bidirectionally sequenced to ensure unimodality and were matched by hand in software. Considering the macro- and microscopic features, we tentatively classified our specimens as *Clitopilusbaronii*. More samples are needed to resolve our uncertainties regarding both the presence of cheilocystidia and the phylogenetic relationship.

#### ﻿ Rhodocybe zijinshanensis

Taxon classificationFungiAgaricalesEntolomataceae

S.P. Jian & X. Chen sp. nov.

329B8FFA-E859-5A05-B6CE-E7AF5E715E5B

857349

**Figure 7.**
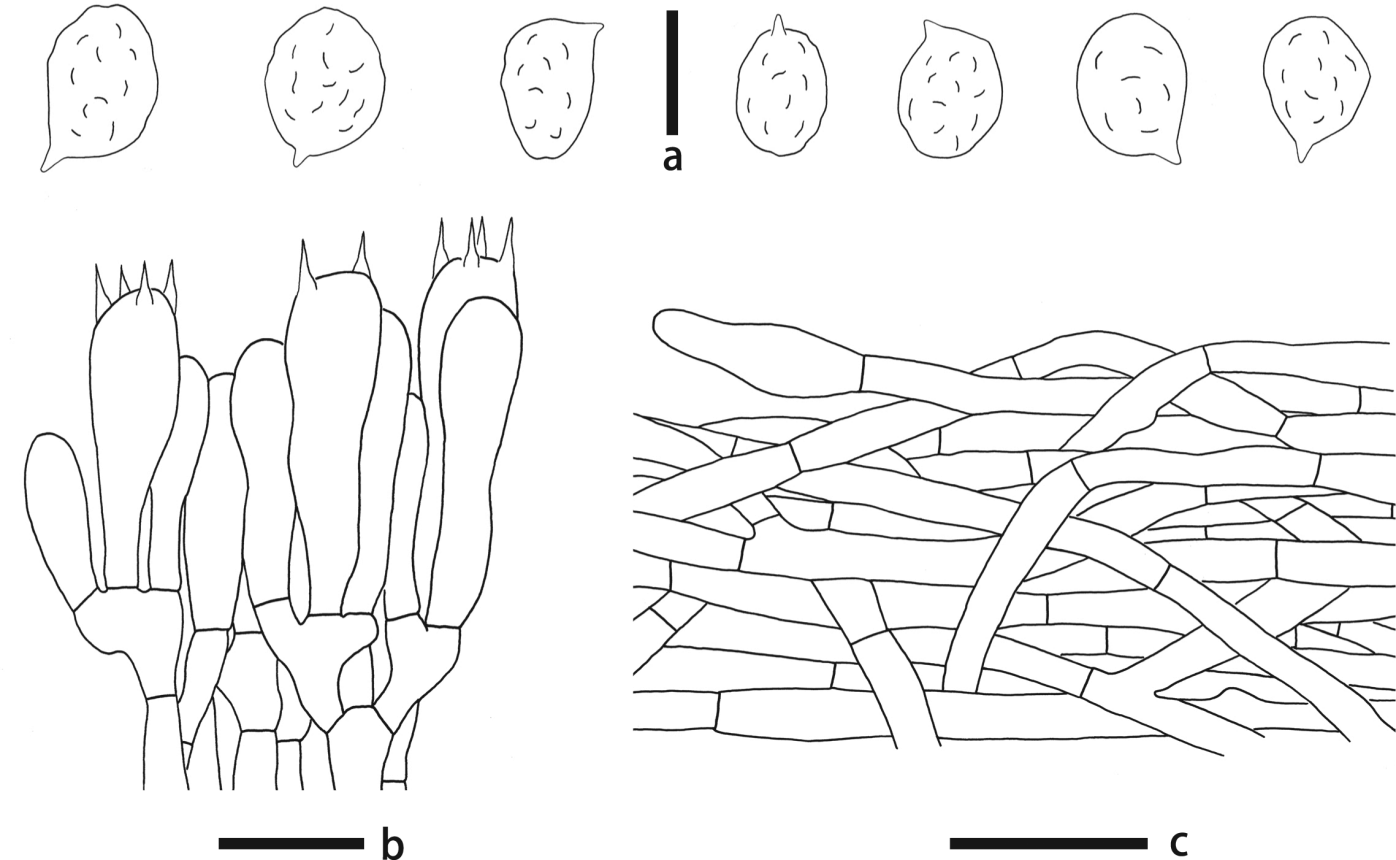
Microscopic features of *Rhodocybezijinshanensis* (KUN-HKAS 145338, holotype) **a** hymenium and subhymenium **b** basidiospores. Scale bars: 5 μm (**a**); 10 μm (**b**); 20 μm (**c**).

Figs 2 , 3i–k , 4g–h , 7a–c

##### Holotype.

China • Jiangsu Province, Nanjing City, Zijinshan, E 118.87, N 32.06, alt. 99 m, solitary on rotten wood, in mixed broadleaf (i.e. *Quercusacutissima*, *Quercusaliena*, *Aphanantheaspera*, *Osmanthusfragrans*, *Liquidambarformosana*, *Photiniaserratifolia* and *Ilexchinensis*) forest, 30 August 2024, collected by X. Chen, CX 664 (KUN-HKAS 145338). GenBank: ITS = PQ793171; LSU = PQ781615; *RPB2* = PQ PQ788400; *TEF1* = PQ788406.

##### Etymology.

“*zijinshanensis*” indicates the source place, where it was located in Nanjing City, China.

##### Diagnosis.

*Rhodocybezijinshanensis* is similar to *R.subasyae*, but differs by its smaller yellow pileus, shorter and more slender stipes and the absence of cheilocystidia.

##### Description.

Basidiomata omphalioid, small size. Pileus 10–15 mm wide, applanate to plano-concave; surface yellow (#eac7a2) over edge and brownish-yellow (#6c3620) over disc, distributing some radiate fibrillose, sometimes hygrophanous; margin slightly inflexed, even or undate; context about 1 mm thick. Lamellae adnate to subdecurrent, yellowish (#cdbead) to greyish-pink (#d1b4a2), dense or crowded, edges entire or undate, sometimes with transverse intervenose, concolorous with lamellae, lamellulae numerous. Stipe 7–19 × 1–2 mm, central to eccentric, cylindrical to tapering downwards, usually concolorous with pileus, densely fine scales dispersed around the top. Odour none.

Basidiospores (4.5) 5–6.5 × 3.5–5 μm, L_m_ × W_m_ = 5.5 (± 0.54) × 4.3 (± 0.31) μm, Q = 1.09–1.55 (Q_avg_ = 1.28 ± 0.11) [41/2/2], hyaline, subglobose, subamygdaliform to broadly ellipsoid in profile view, ellipsoid in face view and minutely, but obviously angular in polar view (7–9 facets in total), undulate-pustulate in all views. Basidia 18.5–32 × 5.5–7.5 μm, clavate, hyaline, 2- or 4-spored; sterigmata up to 5 μm long. Lamellar trama regular, composed of 2.5–10.5 μm in diam., thin-walled, hyaline hyphae. Lamellae edges fertile. Pleurocystidia and cheilocystidia absent. Pileipellis a cutis composed of radially arranged, subregular hyphae, hyphae thin-walled, yellowish, smooth, cylindrical, 3.5–11.5 μm in diam., sometimes with oleiferous hyphae; pileal trama regular, composed of hyaline, thin-walled, cylindrical hyphae with a diameter of 2–11 μm. Stipitipellis a cutis composed of compactly arranged, regular, thin-walled and hyaline hyphae with a diameter of 3.5–9.5 μm; Stipe trama regular, composed of thin-walled and hyaline hyphae with a diameter of 4–10.5 μm. Caulocystidia absent. Clamp connections absent.

##### Ecology and distribution.

Solitary on rotten wood in broad-leaved forest, only found in Jiangsu Province, China, August to October.

##### Additional specimens examined.

China • Jiangsu Province, Nanjing City, Zijinshan, E 118.87, N 32.06, alt. 99 m, solitary on rotten wood, in mixed broadleaf (i.e. *Quercusacutissima*, *Quercusaliena*, *Aphanantheaspera*, *Osmanthusfragrans*, *Liquidambarformosana*, *Photiniaserratifolia* and *Ilexchinensis*) forest, 30 August 2024, collected by X. Chen, CX 665 (KUN-HKAS 145339).

##### Notes.

*Rhodocybezijinshanensis* belongs to R.sect.Rufobrunnea (Fig. 2). Species in this section are characterised by centrally stipitate basidiomata, pilei ranged from pinkish, reddish, brown, tan to fulvous (but never greyish or white), lamellae that are adnexed to adnate or decurrent, the absence of hymenial pseudocystidia and clamp connections (Baroni 1981). *Rhodocybezijinshanensis* is similar to several other species, including *R.asyae* Seslı & Vizzini, *R.subasyae* T. Bau & Y.L. Sun, *R.pseudoalutacea* T.J. Baroni et al. and *R.alutacea* Singer. *Rhodocybeasyae*, first recorded in Turkey, can be differentiated from *R.zijinshanensis* by its relatively larger, smooth pileus (10–30 mm), longer and thicker stipe (25–30 × 2–5 mm) and flexuous cheilocystidia (20–30 × 4–6 μm) (Seslİ and Vizzini 2017). *Rhodocybesubasyae*, a recently described species from Jilin, China, is also similar to *R.zijinshanensis*, but differs in having a smooth pileus, a longer and thicker stipe (22–37 × 5–7 mm), slightly larger basidiospores (Q_avg_ = 1.4), and cheilocystidia measuring 22.4–28.2 × 3.9–6.8 μm (Sun and Bau 2023). For *R.pseudoalutacea*, it was reported from the Dominican Republic, featured by its slightly larger pileus (10–35 mm), slender yet thick stipe (15–50 × 2–6 mm) and pileipellis composed of finely encrusted cylindrical hyphae (Baroni et al. 2020). The last species which resembled *R.zijinshanensis* was *R.alutacea*, found in Florida, USA. It is characterised by the greater pileus size (25–35 mm), a longer stipe (23–35 × 2.5–5.5 mm), and septate, flexuous cheilocystidia (20–35 × 6.5–7 μm) (Singer 1946b; Baroni 1981).

## ﻿Discussion

In this study, we described two new species and documented a new record species in China: *C.parasiticus*, *R.zijinshanensis* and *C.baronii*. For the phylogenetic analysis, we utilised nearly all available sequences for the genera *Clitopilus* and *Rhodocybe*, uploaded by classified references or expert researchers (see Fig. 1). The phylogenetic tree indicates that *C.parasiticus* is closely related to *C.velutinus* T. J. Baroni & Angelini, which was discovered in the Dominican Republic. However, it can be distinguished by its larger pileus (10–25 mm), the existence of an eccentric stipe and larger basidiospores (L_m_ × W_m_ = 8.0 × 5.0 μm) with more longitudinal ridges (10–14) (Baroni et al. 2020). Similarly, *C.baronii* is closer to *C.venososulcatus* Singer, which is occurring only in Florida, USA. Nonetheless, the latter typically exhibits a larger, venose and sulcate pileus (12–23 mm) and slightly larger basidiospores (8–8.5 × 4.5–5 μm) with 6–8 obscurely longitudinal ridges (Singer 1946a). Finally, *R.zijinshanensis* approaches to *R.nuciolens* (Murrill) Singer and *R.gemina* (Paulet) Kuyper & Noordel., but the larger size of their basidiomata (particularly in the pileus and stipe) and the presence of cheilocystidia make them easy to distinguish from the former (Baroni 1981; Seslİ and Vizzini 2017; Vizzini et al. 2018). The similar species of above species are compared in Table 3.

**Table 3.** The comparison of morphological characters amongst *C.parasiticus*, *C.baronii*, *R.zijinshanensis* and similar species.

Taxa	Badisiomata	Pileus	Basidiospores (ridges)	Hymenial cystidia	Habitat	Locality	References
**Clitopilussect.Scyphoides**							
*C.baronii* (Holotype)	Orbicular to conchate or spatulate, sessile	5–40 mm, white to greyish	6.9–8.4 × 4.4–5.5 μm (8–10), Q = 1.68–1.71	Cheilocystidia lageniform	On a decaying trunk of *Quercus* sp.	Italy	Consiglio and Setti (2019)
***C.baronii***	**Conchate, sessile**	**3–15 mm, white to greyish**	**6.5–9.5 × 4–5 μm (8–10), Q = 1.4–1.98**	**None**	**On rotten wood**	**China**	**This study**
*C.daamsii* (Holotype)	Orbicular to conchate, sessile	2–8 mm, white	8–11.5 × 4.8–6.6 μm (6–9), Q = 1.4–2	None	On wood or other fungi	Netherlands	Noordeloos (1984)
*C.fasciculatus* (Holotype)	Fasiculata, sessile	Individual 24 × 20 mm, pale brown	4.7–6.3 × 3.0–3.5 μm (3–6), Q = 1.2–1.85	None	On beds of cultivated mushrooms	Netherlands	Noordeloos (1984)
*C.hobsonii* (Holotype)	Orbicular or slightly reniform, sessile	5–18 mm, white to pale greyish	6.5–9 × 4–5.5 μm (7–12), Q = 1.2–2	None	On plant debris or herbaceous stems	Britain	Orton (1960)
***C.parasiticus* (Holotype)**	**Conchate, sessile**	**2–8 mm, whitish to chalk white**	**5.5–8.5 × 3.5–5 μm (7–9), Q = 1.2–1.9**	**None**	**On soil, rotten wood and leaves of plants**	**China**	**This study**
*C.passeckerianus* (Holotype)	Reniform or resembling an ear, sessile	8–40 mm, white	7–9 × 4–5 μm (7–12), Q = 1.45–2.25	None	On mushroom-beds	Europe	Pilát (1935)
*C.pinsitus* (Holotype)	Spatulate, semi-cicular, sessile	15–40 mm, white to pale ochre	7–9 × 4.6–5.3 μm (7–8)	None	On trunk of *Quercus* sp.	Sweden	Josserand (1937)
*C.velutinus* (Holotype)	Clitocyboid	10–25 mm, pure white	7–9 × 5–6 μm (7–8), Q = 1.27–1.8	None	On soil	Dominican Republic	Baroni et al. (2020)
*C.venososulcatus* (Holotype)	Pleurotoid, sessile or sub sessile	12–23 mm, pallid white	8–8.5 × 4.5–5 μm (6–8)	None	On trunks or logs of Ficus aurea	USA	Singer (1946a)
**Rhodocybesect.Rufobrunnea**							
*R.alutacea* (Holotype)	25–35 mm, yellowish, hygrophanous	23–35 × 2.5–5.5 mm, subequal	5.8–7.5 × 3.5–5 μm (7–9)	Cheilocystidia	On sandy soil and fallen leaves	USA	Singer (1946b)
*R.asyae* (Holotype)	10–30 mm, salmon pink	25–30 × 2–5 mm, tapering	5–7 × 4–5 μm, Q = 1.1–1.4	Cheilocystidia	On the grass	Turkey	Seslİ and Vizzini (2017)
*R.gemina*	15–80 mm, reddish incarnate	25–50 × 3–15 mm, subequal	5–6.5 × 4–5 μm	Cheilocystidia	On humus	Europe	Baroni (1981)
*R.nuciolens*	10–60 mm, pinkish cinnamon, hygrophanous	35–80 × 2–9 mm, equal	5.5–8 × 4–5 μm	Cheilocystidia	On humus, sandy soil or decaying wood	USA	Baroni (1981)
*R.pseudoalutacea* (Holotype)	10–35 mm, brown or brownish orange, hygrophanous	15–50 × 2–6 mm, equal or enlarged downwards	5.5–7 × 4–5 μm (7–10), Q = 1.2–1.6	None	On decaying humus or woody debris	Dominican Republic	Baroni et al. (2020)
*R.subasyae* (Holotype)	19–25 mm, beige red	22–37 × 5–7 mm, cylindrical	5.4–6.8 × 3.9–4.9 μm (6–8), Q = 1.2–1.6	Cheilocystidia	On sandy soil	China	Sun and Bau (2023)
***R.zijinshanensis* (Holotype)**	**10–15 mm, yellow, hygrophanous**	**7–19 × 1–2 mm, cylindrical to tapering**	**5–6.5 × 3.5–5 μm (7–9), Q = 1.09–1.55**	**None**	**On rotten wood**	**China**	**This study**

In the family Entolomataceae Kotl. & Pouzar, there are over 1500 described species worldwide (Co-David et al. 2009; Baroni and Matheny 2011; Karstedt et al. 2019). However, only a few species exhibit mycoparasitic capabilities, like *Entolomaabortivum* (Berk. & M.A. Curtis) Donk, *E.parasiticum* (Quél.) Kreisel, *E.pseudoparasiticum* Noordel. and *Rhodophanastangliana* (Bresinsky & Pfaff) Vizzini (Noordeloos 1988; Læssøe and Rosendahl 1994; Czederpiltz et al. 2001). Thereinto, *Entolomaabortivum* is frequently reported to co-occur with *Armillaria* (Fr.) Staude, leading to the hypothesis that *Armillaria* attacks and parasitises the basidiomata of *Entolomaabortivum* (Watling 1974). On the contrary, Czederpiltz et al. (2001) demonstrated that *E.abortivum* can actually abort the growth of Armillaria in culture media. Furthermore, Koch and Herr (2021) explained this phenomenon using transcriptomics.

Notably, some species, such as *E.clypeatum* (L.) P. Kumm., *E.niphoides* Romagn. ex Noordel., *E.saepium* (Noulet & Dass.) Richon & Roze and *E.sericeoides* (J.E. Lange) Noordel., have been reported to associate with rosaceous woody plants. However, these species are more likely to be detrimental to roots rather than forming typical mycorrhizae (Agerer and Waller 1993; Gryndler et al. 2010; Shishikura et al. 2020). In the *Rhodocybe-Clitopilus* clade, *C.daamsii* has been observed growing on *Hydnoporiatabacina* (Sowerby) Spirin et al. (previously classified as *Hymenochaetetabacina* (Sowerby) Lév.), while *C.passeckerianu* and *C.fasciculatus* have been associated with the growing-beds of cultivated *Agaricus* L. (Noordeloos 1984; Noordeloos 1988; Noordeloos 1993), although Singer questioned the mycoparasitic behaviour of *C.passeckerianu* (Singer and Harris 1987).

To investigate the saprophytic and biotrophic abilities of *C.parasiticus*, we carefully examined different specimens to identify the discrepancies between various hosts and growth on soil. The results are presented in Table 4. We found that the basidiospores from specimens KUN-HKAS 145335 and 145337, which were collected from the leaves of *Oplismenus* sp. and *Dryopteris* sp., respectively, showed no significant differences. However, there was an obvious difference with specimen KUN-HKAS 145336, where the basidiospores of *C.parasiticus* growing on soil were larger than those from specimens growing on plant leaves. Larger basidiospores often indicate more robust growth of basidiomata (Kauserud et al. 2011; Halbwachs et al. 2017), suggesting that this species may be better suited to a soil habitat than to a biotrophic lifestyle.

**Table 4.** The intraspecies comparison of *C.parasiticus* in morphological characters and microenvironment.

Taxa	Voucher specimen	Pileus	Basidiospores (ridges)	Crystals in pileipellis	Habitate	Temp. (°C)	Prec. (mm/d)
*C.parasiticus*	KUN-HKAS145335 (CX628)	2–8 mm	5.5–7.0 × 4–5.5 μm, L_m_ × W_m_ = 6.3 (± 0.47) × 4.24 (± 0.35) μm, Q = 1.20–1.84 (Q_avg_ = 1.49 ± 0.13) (8–9) [63/3/1]	None	On leaves of *Oplismenusundulatifolius*	29.04	5.58
*C.parasiticus* (Holotype)	KUN-HKAS145336 (CX966)	3–7 mm	6.0–8.5 × 4–5 μm, L_m_ × W_m_ = 7.06 (± 0.6) × 4.40 (± 0.30) μm, Q = 1.40–1.81 (Q_avg_ = 1.61 ± 0.10) (7–8) [62/3/1]	Present	On soil	31.25	4.43
*C.parasiticus*	KUN-HKAS145337 (CX967)	3–5.5 mm	5.5–7.5 × 3.5–5 μm, L_m_ × W_m_ = 6.33 (± 0.50) × 4.09 (± 0.28) μm, Q = 1.20–1.90 (Q_avg_ = 1.55 ± 0.13) (7–9) [61/3/1]	Present	On leaves of *Dryopteris* sp.	31	4.42

Notes: Temp./Prec. means the average temperatures/precipitations one week before and one week after the date of collection.

Furthermore, the average temperature over a fortnight in 2024 was slightly higher than in 2023, while the average precipitation during the same period was slightly lower in 2024 compared to 2023. These subtle discrepancies could influence the nutritional mode and even the choice of parasitic host. Admittedly, our judgement that this species is biotrophic on the basis of only two collections from different plant leaves, is not entirely rigorous. More experiments, including physiological and genomic analyses, are necessary for a comprehensive assessment.

## Supplementary Material

XML Treatment for Clitopilus parasiticus

XML Treatment for Clitopilus baronii

XML Treatment for Rhodocybe zijinshanensis

## ﻿Additional information

### Author ORCIDs

Sipeng Jian https://orcid.org/0000-0002-2055-3169

Xia Chen https://orcid.org/0009-0002-1239-1865

### Data availability

In this study, DNA sequences have been deposited in GenBank. Specimens were placed at Herbarium of Kunming Institute of Botany, Chinese Academy of Sciences (KUN-HKAS).
